# Tendon Organoids Enable Functional Tendon Rejuvenation Through ALKBH5‐Dependent RNA Demethylation

**DOI:** 10.1002/advs.202508376

**Published:** 2026-01-28

**Authors:** Tian Qin, Aini Pan, Zhuoning Miao, Yanyan Zhao, Xianan Mo, Heng Sun, Chunmei Fan, Junyu Guo, Bingbing Wu, Weiliang Shen, Qiangqiang Zheng, Jun Lu, Xi Jiang, Zi Yin, Xiao Chen

**Affiliations:** ^1^ Department of Sports Medicine & Orthopedic Surgery the Second Affiliated Hospital and Liangzhu Laboratory Zhejiang University School of Medicine Hangzhou China; ^2^ Department of Orthopedic Surgery of Sir Run Shaw Hospital and Liangzhu Laboratory Zhejiang University School of Medicine Hangzhou China; ^3^ Dr. Li Dak Sum & Yip Yio Chin Center for Stem Cells and Regenerative Medicine Zhejiang University School of Medicine Hangzhou China; ^4^ Zhejiang Key Laboratory of Motor System Disease Precision Research and Therapy Hangzhou China; ^5^ Zhejiang University‐University of Edinburgh Institute Zhejiang University School of Medicine Haining China; ^6^ Medical 3D Printing Center, Orthopedic Institute Department of Orthopedic Surgery The First Affiliated Hospital School of Basic Medical Sciences Suzhou Medical College Soochow University Suzhou Jiangsu P. R. China; ^7^ Key Laboratory of Novel Targets and Drug Study For Neural Repair of Zhejiang Province Department of Clinical Medicine School of Medicine Hangzhou City University Hangzhou Zhejiang China; ^8^ The Fourth Affiliated Hospital International Institutes of Medicine Zhejiang University School of Medicine Yiwu China; ^9^ Department of Pharmacology and Bone Marrow Transplantation Center of the First Affiliated Hospital Zhejiang University School of Medicine Hangzhou Zhejiang China; ^10^ Institute of Hematology Zhejiang University and Zhejiang Engineering Laboratory for Stem Cell and Immunotherapy Hangzhou Zhejiang China; ^11^ Liangzhu Laboratory Zhejiang University Medical Center Hangzhou Zhejiang China

**Keywords:** ALKBH5, mRNA demethylation, tendon organoids, tendon regeneration, tendon stem/progenitor cells

## Abstract

Adult tendon injuries pose a major clinical challenge due to limited self‐repair capacity, resulting in suboptimal regeneration. Although tendon stem/progenitor cells (TSPCs) are pivotal for tendon engineering, achieving microstructure and functional regeneration remains challenging. Organoids boost tissue regeneration post‐transplantation in multiple organs. however, tendon‐specific organoids with stable phenotype and regenerative capacity are still lacking. Since fetal tendons possess strong regenerative capabilities, the construction of fetal‐like tendon organoids is crucial for promoting tendon regeneration. In this study, we generated fetal‐like tendon organoids (FT organoids) from adult TSPCs using a serum‐free 3D culture system that recapitulated the in vivo microenvironment. These organoids exhibited robust phenotypic restoration and enhanced tenogenic potential, with a gene expression profile resembling fetal tendon development. Notably, transplantation experiments demonstrated functional regeneration of organized tendon collagen matrices in vivo. Furthermore, the mRNA demethylase ALKBH5 plays a critical role in activating key regulatory networks for tendon regeneration via the TGF‐β signaling pathway. These findings provided compelling evidence that FT organoids represent a promising strategy for tendon collagen microstructure regeneration and highlights their promising clinical translation potential.

## Graphical abstract

FT organoids reverse the aged phenotype of tendon cells, reinstating a fetal‐like state. This breakthrough establishes a potent cell source for tendon tissue engineering, effectively advancing regenerative medicine.

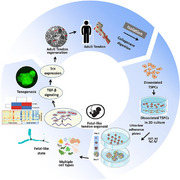

## 1 Introduction

Connective tissue is a type of tissue found throughout the body that serves a variety of important functions. Tendons, as a distinctive type of connective tissue, function to connect muscles to bones and enable the transmission of forces, thereby playing a pivotal role in the body's movement. However, the regenerative capacity of adult tendons is limited, and their repair after injury faces challenges such as tendon adhesion, diminished collagen fiber size, compromised mechanical properties, and the risk of recurrent fractures, which persist in clinical treatments [1, 2]. Tissue engineering has emerged as a pivotal strategy for tendon repair and regeneration, as it aims to create implantable artificial tissues to restore the structure and function of damaged tendons. Tendon stem/progenitor cells (TSPCs) [3], as crucial seed cells in tendon tissue engineering, possess pluripotency, self‐renewal capability, and express high levels of tendon‐specific proteins such as collagen I, and tenascin‐C. Nevertheless, the loss of tendon stem cell phenotype during in vitro culture presents a major challenge in tendon tissue engineering [4, 5]. For instance, the expression of pivotal tendon genes, such as Scleraxis (Scx), gradually becomes suppressed when TSPCs are cultivated in a conventional 2D plate environment, leading to functional alterations [6, 7, 8].

Advancements in 3D culture techniques have allowed embryonic and adult stem cells to form organoids, which mirror the structure and function of native organs. Organoids, as a promising alternative to traditional 2D cultures, enhance physiological relevance by replicating cell interactions, matrix growth, and hypoxic conditions, closely mimicking their natural counterparts [9, 10]. To date, numerous organoids for various tissues, such as the intestine [11], gut [12], brain [13], retina [14], liver [15], bone [16, 17], and cartilage [18], displaying an improved functionality. Nevertheless, at present, there is a lack of universally acknowledged effective and stable methods for the construction of organoids derived from TSPCs. Therefore, we aim to construct TSPCs‐derived FT organoids, preserving their native phenotype and fetal‐like regenerative capacity. By leveraging the transplantation potential of these organoids, we seek to address the limitations in adult tendon regeneration and facilitate the repair of injured tendons in adults.

It has been reported that the 3D culturing microenvironment induces epigenetic modifications in cells of multiple lineages, playing a pivotal role in regulating the processes of stem cell differentiation and reprogramming specifically within organoids [19, 20, 21, 22, 23, 24]. Studies have highlighted the impact of RNA demethylation on stem cell behavior in organoids [25]. For instance, the RNA demethylase ALKBH5 was reported to influence breast cancer stem cell cultured in a 3D microenvironment [26]. The m6A methyltransferase METTL14 has also been found to regulate intestinal organoid growth [27]. Therefore, gaining an understanding of the impact of mRNA modifications on organoids is imperative for unraveling the mechanisms underlying their phenotypic maintenance and manipulating their differentiation.

In this study, we effectively constructed TSPCs‐derived FT organoids and utilized single‐cell transcriptome analysis to reveal the intricate functional cell subsets and the fetal‐like properties within these FT organoids. Our innovative approach addressed the issues of TSPCs phenotype loss observed in 2D culture. Notably, the FT organoids exhibited enhanced stemness and tenogenesis capacities in vitro and promoted tendon regeneration after in vivo transplantation, making them highly promising seed cells for tendon tissue engineering. Furthermore, we demonstrated the pivotal role of the mRNA demethylase ALKBH5 in regulating the phenotype of TSPC through TGF‐β signaling pathway, shedding light on its importance in tendon development.

## 2 Results

### 2.1 The Fetal‐Like Tendon Organoids Exhibited a Cellular Composition Analogous to In Vivo Tendons as Well as a Fetal‐Like Cell state

**FIGURE 1:**
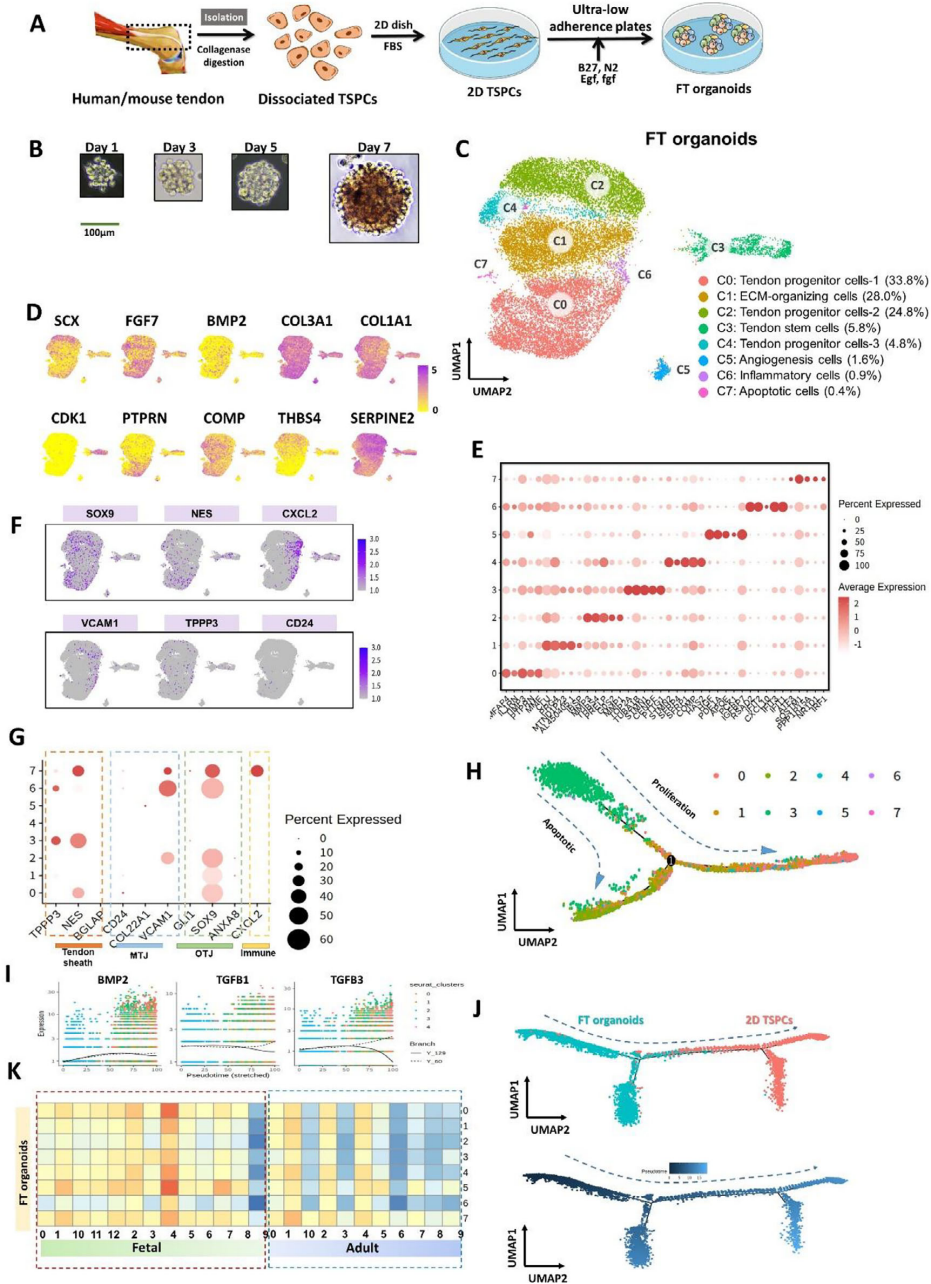
The FT tendon organoids possessed cell types analogous to those in vivo tendons and exhibit characteristics resembling fetal tendons. (A) Schematic of the experimental protocol. (B) Brightfield (BF) of FT organoids from 1 day to 7 days. Scale bars, 100 µm. (C) UMAP visualization of single cells from FT organoid cultures, where individual points correspond to single cells. Cells are colored by annotated cell types. (D) Expression patterns of well‐known tendon development‐associated markers were projected onto the UMAP. The colors from yellow to purple represent expression levels from low to high. I Dot plot of marker genes of each cluster. (F) Expression patterns of tendon tissue‐composition markers were projected onto the UMAP. The colors from gray to purple represent expression levels from low to high. (G) Dot plot of tendon tissue‐composition markers. (H) Pseudo‐temporal trajectory of cells from FT organoids by Monocle. The color represented clusters consistent with in C. (I) The expression patterns of BMP2, TGFB1 and TGFB3 on the pseudo‐temporal trajectory colored by clusters in A. (J) Pseudo‐temporal trajectory of cells from FT organoids and 2D TSPCs combined by Monocle. The color represented FT Organoids and 2D TSPCs (upper) and pseudo‐time (below). (K) Heatmaps of correlation coefficients between FT organoids and the expression of each cluster of fetal and adult tendons in FigureS1B.

To construct the FT organoids, the TSPCs were isolated from adult human tendon, and initially cultured and expanded in a 2D environment using fetal bovine serum (FBS) according to traditional cultivation methods. Subsequently, we employ a low‐adhesion culture system to cultivate the digested tendon stem cells in a specific tendon organoid culture medium (see Methods) (**Figure** **1**A). TSPCs aggregated into spheres with a diameter of approximately 100 µm on Day 1 (1 day after the initiation of suspension culturing) and differentiated, assembling into spherical organoids with a diameter of about 140 to 180 µm by day 7 in our FT culturing medium (Figure 1B, Figure S1A,B). As a control, we also subjected the same batch of primary TSPCs to 2D culture conditions (DMEM, serum). Both FT organoids and 2D condition TSPCs exhibited similar surface marker expression profiles (Figure S1C). To gain insight into the cell populations in the FT organoids, we employed single‐cell sequencing technology [28, 29] to explore the cellular heterogeneity within our FT organoids. We isolated and sequenced mRNA from a total of 20 359 cells obtained from day 7 organoids (TSPCs originated from three individual patients). These cells were identified as the tendon stem cell clusters, tendon progenitor cells 1 and 2 clusters, ECM clusters, immune clusters, angiogenic clusters based on their marker genes and GO functional analysis (Figure 1C–E), and validated by Immunofluorescence staining (Figure S1D). Pseudo‐temporal analysis using Monocle was performed on the populated cells, revealing two trajectories (proliferation and apoptosis) originating from the cycling cluster (Figure 1H). Key tendon differentiation genes, such as *BMP2*, *TGFB1*, and *TGFB3*, were up‐regulated in the proliferation trajectory and down‐regulated in the apoptosis trajectory (Figure 1I). The FT organoid contained distinct cell types that present in natural tendon tissues in vivo, including tendon sheath, muscle‐tendon junction (MTJ), bone‐tendon junction (BTJ), and immune system cells (Figure 1F,G). To investigate the similarities between our organoids and in vivo tendon tissue cells, we also separately conducted single‐cell transcriptome profiling on human adult and fetal tendon tissues. By clustering these cells, we identified that both adult and fetal tendon cells encompass clusters of tendon stem cells, tendon progenitor cells, ECM assembly cells, immune‐related cells, and angiogenic populations, which were consistent with the clusters within the organoids. Notably, we detected more tendon stem cell clusters in fetal tendon cells, specifically tendon stem cell cluster 1 marked by the gene CDK1, tendon stem cell cluster 2 marked by the gene SCX, and tendon stem cell cluster 3 marked by the gene CD55 (Figure S1E). Through the correlation analysis, we found that FT organoids clusters exhibited higher resemblance with the fetal tendon clusters compared with adult tendons (Figure 1K; Figure S1F). These results further validated that our organoid mimics a fetal‐stage tendon state.

We also compared the transcriptome differences between FT organoids and 2D TSPCs at the single‐cell level (Figure S2A). We identified these clusters through the marker genes combining with GO analysis results of each cluster (Figure S2B–F). In comparison, the expression levels of tendon development‐related genes such as *SCX*, *COL3A1*, and *PTPRN* are higher in almost all clusters of the organoids than in 2D TSPCs (Figure S2D). Pseudo‐temporal analysis conducted with Monocle revealed that FT organoids are positioned at an early stage along the pseudo‐timeline in comparison to 2D TSPCs (Figure 1J). These results suggested that the FT organoids reverted the 2D TSPCs derived from adult tendon to a more primitive state. This functionality remains effective even in TSPCs derived‐from older patients (Figure S1G).

### 2.2 TSPCs‐derived FT Organoids Exhibited Superior Stemness and Tenogenesis In Vitro

**FIGURE 2:**
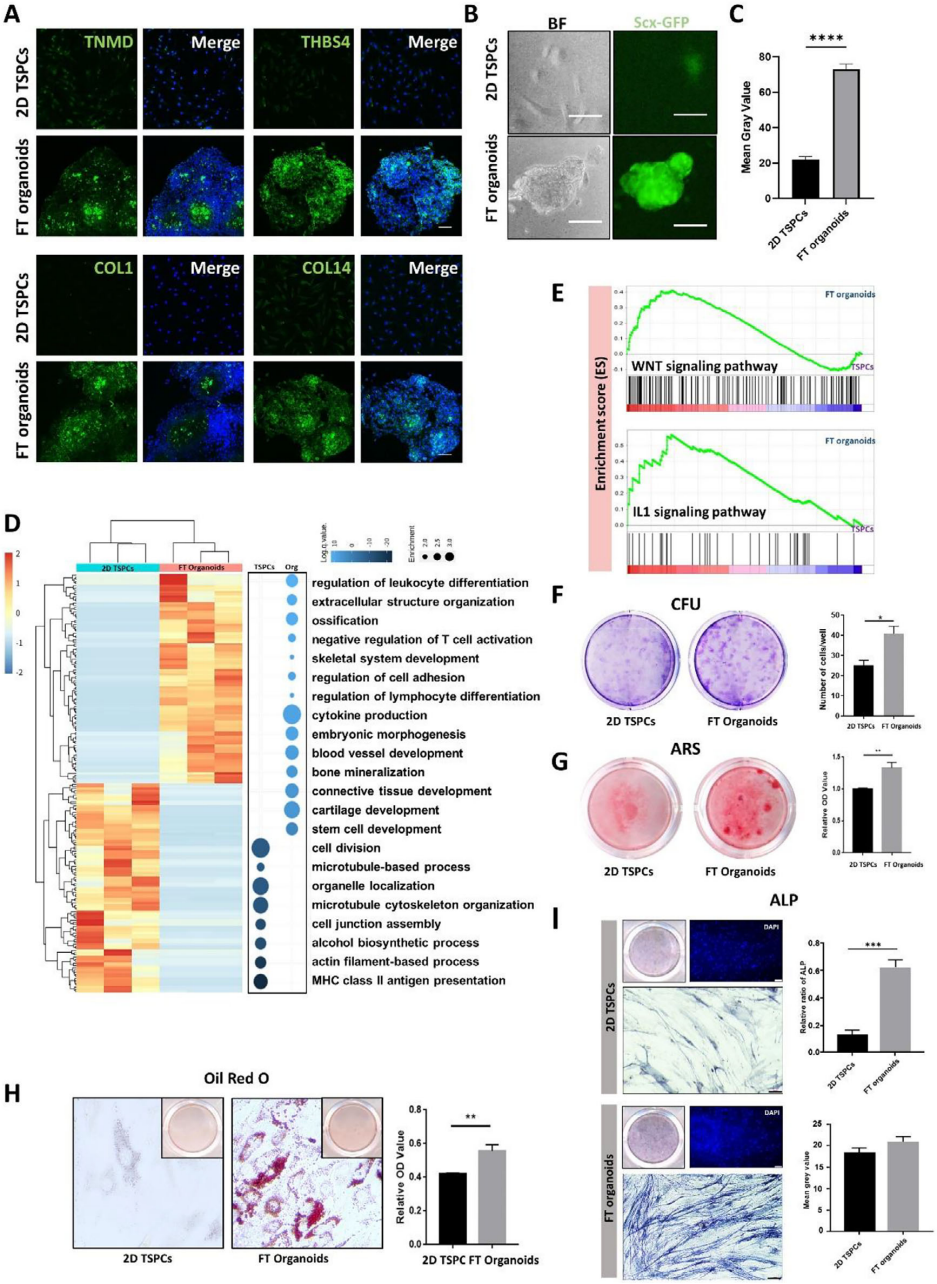
The FT organoids exhibited enhanced of genes related to tendon‐development genes expression, as well as increased stemness and multilineage differentiation capabilities. (A) Protein expressions of tendon markers (TNMD, THBS4, COL1, and COL14) of FT organoids and 2D TSPCs. Nuclei were stained with DAPI. Scale bars, 50 µm. (B) Bright‐field (BF) and fluorescence images of FT organoids and 2D TSPCs derived from Scx‐GFP mice. Scale bar, 200 µm. (C) Mean gray value of SCX fluorescence intensity in FT organoids and 2D TSPCs. ****, p<0.0001 (Student's t test). (D) Heatmap of differentially expressed genes and GO analysis of differential genes from RNA‐seq results of organoids and 2D TSPCs. (E) GSEA analysis of RNA‐seq results of FT organoids vs 2D TSPCs. (F) CFU assay showing the self‐renewal of hTSPCs. Clones were visualized by methyl violet staining. The number of initial cells per well was 200 (left). The number of clones per well was counted (right) (n = 3). (G) ARS staining (left) and activity assay. (H) Oil red O staining (left) and quantification of accumulated lipid vacuoles (right). Relative OD values at 490 nm. OD value at 520 nm. (n = 3) **, *p* < 0.01 (Student's t test). (I) ALP staining (left), ALP activity assay (right, above), and mean gray value (right, below) for mineralization (n = 3). ***, *p* < 0.001 (Student's t test). Scale bars and 200 µm.

Tendon‐associated proteins TNMD, THBS4, COL1, COL14, and Tenascin‐C expressed higher in FT organoids than in 2D TSPCs (Figures 2A, S3A, B). Expression of stress‐responsive and bone‐development proteins, including IL1B, BMP2, EGR1, and RUNX2, was also elevated in the organoids (Figure S3C). To visualize the expression levels of the TSPCs marker gene *Scx*, we used TSPCs isolated from *scx*‐GFP mice and found that it expressed significantly higher in FT organoids compared to 2D TSPCs (Figure 2B). To examine their transcriptome profile differences, we performed bulk RNA sequencing (RNA‐seq) on FT organoids and 2D TSPCs (TSPCs originated from three individual patients). Comparing with 2D cultured samples, the results confirmed the up‐regulated expression of tenogenic marker genes, such as HOXA11, BMP2, EGR1, and SCX, as well as down‐regulated expression of markers favoring tendon maturation, including COL14A1, COL1A1 in FT organoids (Figure S2G). The expressions of chondrocyte‐associated genes MMP13, COMP, ACAN, SOX9, and VEGFA, and adipocyte‐associated genes LPL, PPARA, RILPL1, and CFDP1 were up‐regulated in the FT organoids (Figure S2H,I). The differential expression (DE) analysis and gene ontology (GO) analysis were carried out for characterization of the expression profiles (Figure 2D). GO results showed that FT organoids displayed stronger stem cell development and enhanced multi‐lineage differentiation ability, including blood vessel generation, bone mineralization, lymphocyte differentiation, leukocyte differentiation and skeletal system development. The organoids also exhibit the ability to regulate cell‐cell adhesion, indicating that the increased cell contact for the organoids, thereby facilitating stronger intercellular communication (Figure 2D). Meanwhile, gene set enrichment analysis (GSEA) demonstrated that the regenerative‐related signaling pathways such as WNT and IL1 signaling pathways were enhanced in FT organoids than those in 2D TSPCs (Figure 2E). The tendon‐associated gene set was also selectively examined and showed higher expression in FT organoids than in 2D TSPCs (Figure S3D).

We next examined whether FT organoids could increase TSPCs’ stemness in vitro. CFU assays seeded with 100, 200, and 400 cells per well revealed that FT organoids markedly increased the self‐renewal capacity of TSPCs compared with 2D (Figure 2F, Figure S3E). The multi‐differentiation potential of TSPCs toward adipogenic and osteogenic lineages was assessed as described previously [4]. The competent osteogenic differentiation ability of FT organoids was evidenced by stronger alkaline phosphatase (ALP) staining activity and Alizarin Red S (ARS) staining activity (Figure 2G,I). The increased intensity of oil red O staining demonstrated the enhanced adipogenic potential of FT organoids (Figure 2H). These results indicated that FT organoids can maintain TSPCs’ stemness state with their self‐renewal capacity and multi‐differentiation potential.

**FIGURE 3:**
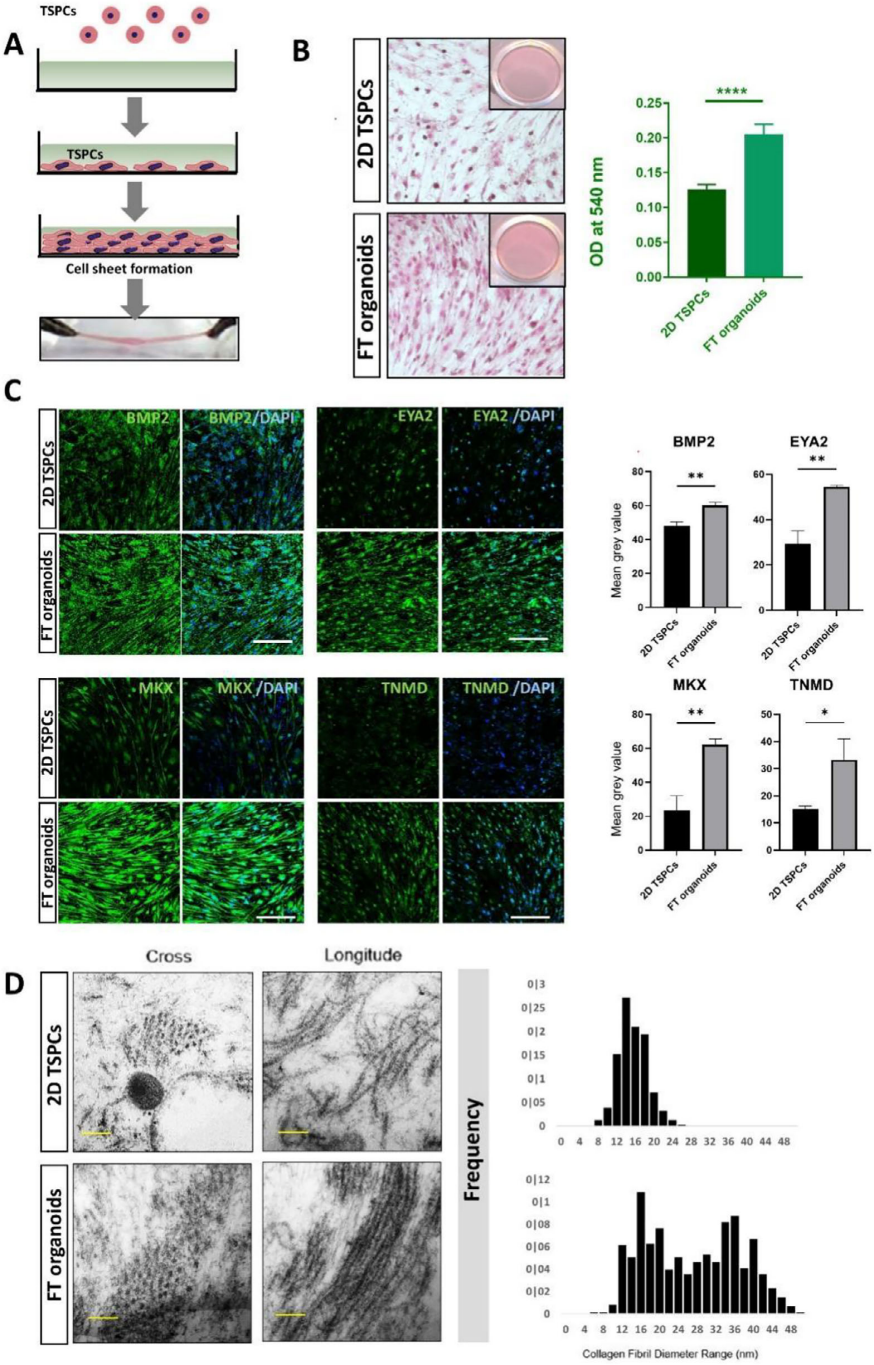
Tenogenesis differentiation potential of FT organoids. (A) A multilayered cell sheet was detached from the substratum by applying a small roll‐up force after culturing in Dulbecco's modified Eagle's medium supplemented with ascorbic acid for 14 days. (B) Sirius red staining for collagen deposition evaluation in TSPCs at 14 days after culturing in Dulbecco's modified Eagle's medium supplemented with ascorbic acid (left), and quantification data of Sirius red staining (right). (C) IF showed the protein expressions of tendon‐associated proteins MKX, TNMD, BMP2 and EYA2 of FT organoids and 2D TSPCs groups. Nuclei were stained with DAPI. Mean gray value of fluorescence intensity in FT organoids and 2D TSPCs. (D) Transmission electron micrographs of cross‐ and longitudinal sections of collagen fibrils in cell‐sheets from FT organoids and 2D TSPCs groups (left); the diameter range of collagen fibrils in the FT organoids groups were significantly greater than in the 2D TSPCs group (right). All data are presented as the mean ± SD. *, *p* < 0.05; **, *p* < 0.01, ***p < 0.001 or ****p < 0.0001. (Student's t test).

In our investigation into the tenogenesis of FT organoids, we examined the in vitro cell‐sheet‐forming capacity of both FT organoids and 2D TSPCs. In the presence of ascorbic acid, TSPCs formed coherent cell sheets within 14 days after attaining confluence (**Figure** **3**A). Sirius red staining was conducted to evaluate the extracellular matrix, especially collagen secretion and production, which is the major component in tendon tissue. The intensity of Sirius red staining was significantly higher in FT organoids than in 2D TSPCs group (Figure 3B). Furthermore, immunofluorescence staining indicated that the tendon‐associated protein levels of MKX, TNMD, BMP2, and EYA2 were upregulated in FT organoids forming cell‐sheet (Figure 3C). We also calculated the range of collagen fibril diameters in these two groups and found that most of these ranged from 8 to 48 nm in the FT organoids, which were significantly larger than either the control group (8–24 nm) (Figure 3D). Taken together, these data obtained from our study suggests that FT organoids exhibit enhanced tenogenic differentiation compared to 2D TSPCs.

### 2.3 FT Organoids Demonstrated a Significant Capacity to Promote In Vivo Regeneration

**FIGURE 4:**
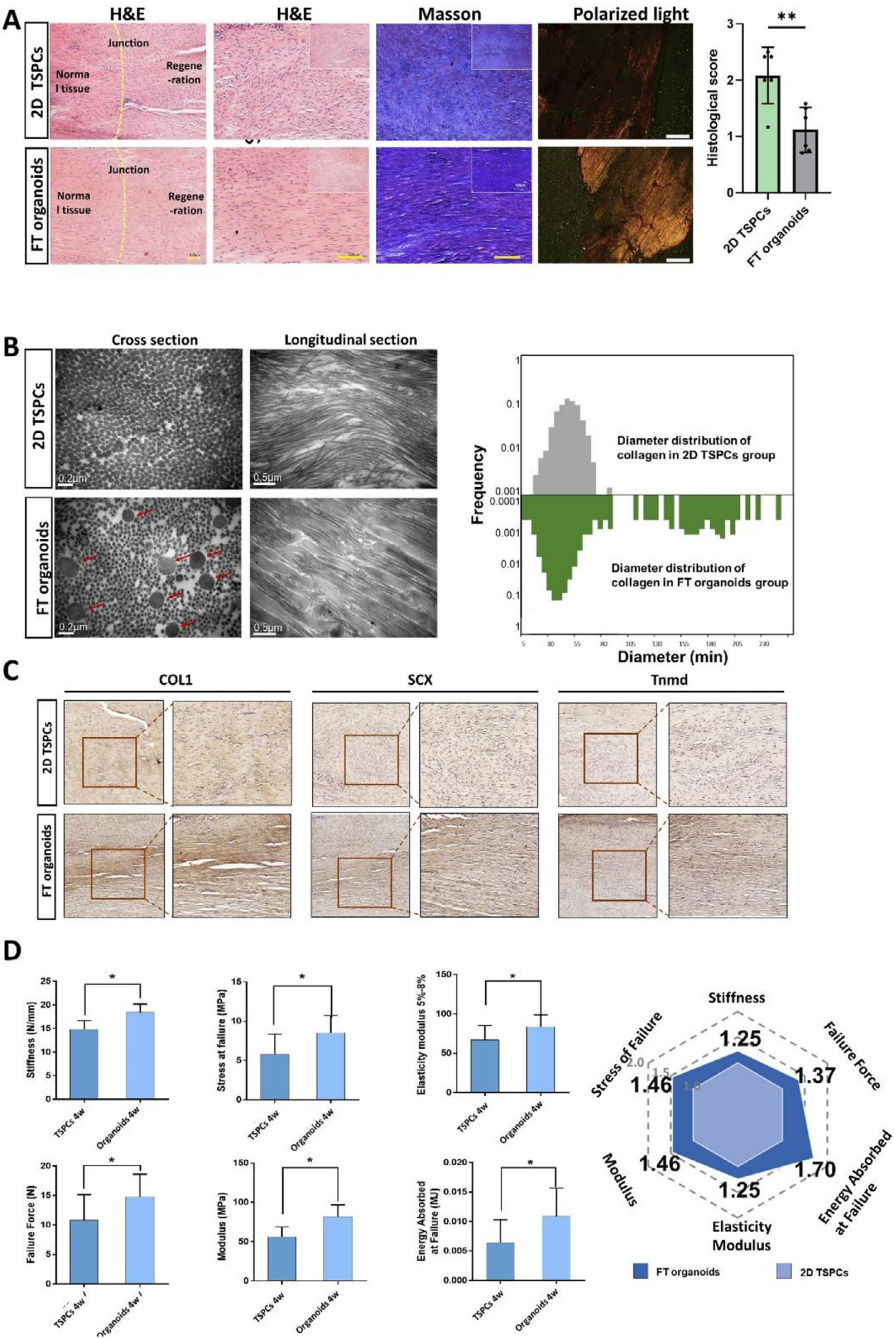
The in vivo reparative potential of FT organoid transplantation for rat's tendon injuries. (A) Morphology of the repaired tissue site along the axis of the patellar tendon in the FT organoids and 2D TSPCs groups. H&E staining and polarized light microscopy showing the collagen fibrils at 4 weeks after implantation. The maturation of repaired tendon was assessed by histology scoring. Masson's trichrome staining showing the deposited collagen at the repaired tissue site and quantitative analyses of the collagen content. Histology scoring of the repaired tendons (n = 6). (B) Transmission electron micrographs of cross‐sections of collagen fibrils in the FT organoids and 2D TSPCs groups from repaired tendons at 4 weeks after implantation. The average diameters and range of diameters of collagen fibrils in the organoids group were significantly larger than in 2D TSPCs group. (C) Immunohistochemical staining of tendon regeneration‐related proteins in the repaired patellar tendon. (D) The biomechanical properties of the repaired tendon (stiffness, failure force, energy absorbed at failure, stress at failure, and modulus) in the FT organoids and 2D TSPCs groups at 4 weeks after implantation. All data are presented as the mean ± SD. *, *p* < 0.05; **, *p* < 0.01 (Student's t test). Scale bars 100 µm in (A), 200 nm in (B). Abbreviation: H&E, hematoxylin and eosin.

Next, we evaluated the tendon regeneration capacity of FT organoids in vivo. Samples were collected and analyzed at 1, 2, or 4 weeks after implantation. Histological examination of the repaired tendons from the FT organoids group revealed the presence of a mature tendon structure at 4 weeks post‐surgery. The post‐transplantation cell survival rate reached approximately 80% in all groups (Figure S4C). In stark contrast to the loosely arranged soft tissues observed in the 2D group, the repaired tendon derived from the FT organoids exhibited a denser composition characterized by distinct crimped patterns of collagen fibrils (**Figure** **4**A). The formation of continuous collagen fibers at the junction site was confirmed under polarized light (Figure 4A). The FT organoids group showed better histological morphology from 1 to 4 weeks, indicating that the injured tissues regenerated gradually over time (Figure 4A; Figure S4A). Additionally, the Masson trichrome staining demonstrated that more collagen was deposited in the FT organoids group when compared to the 2D group. To correlate the histological morphology with the ultrastructure of repaired tendons, samples were examined by transmission electron microscopy (TEM). The FT organoids group illustrated larger fibril diameter than in the 2D group, as shown by the distribution of fibrils diameters (Figure 4B). In the FT organoids group, there was a 2.5% presence of bulky diameter collagen fibers ranging from 110 to 260 nm, which were absent in the 2D group at 4 weeks postoperatively (Figure 4B). Immunohistochemistry staining demonstrated higher expression levels of tendon development‐related proteins COL1, SCX, and TNMD in the FT organoid group compared to the 2D group at 4 weeks postoperatively (Figure 4C, Figure S4D). Immunofluorescence staining further confirmed that the expression of SCX was significantly upregulated in the FT organoid group at 1 and 2 weeks after surgery (Figure S4B). Finally, the mechanical properties (stiffness, failure force, energy absorbed at failure, modulus, elasticity of modulus 5%–8%, and stress at failure) of the FT organoid group, evaluated using an Instron mechanical testing machine, exhibited a significant improvement compared to the 2D group (Figure 4D, Figure S4E). These results indicated that the transplanted FT organoids served as highly potent seed cells in tendon tissue engineering, demonstrating a superior capacity to promote the regeneration of injured tendons.

### 2.4 Epigenetic Modifications of the FT Organoids by Demethylase ALKBH5

**FIGURE 5:**
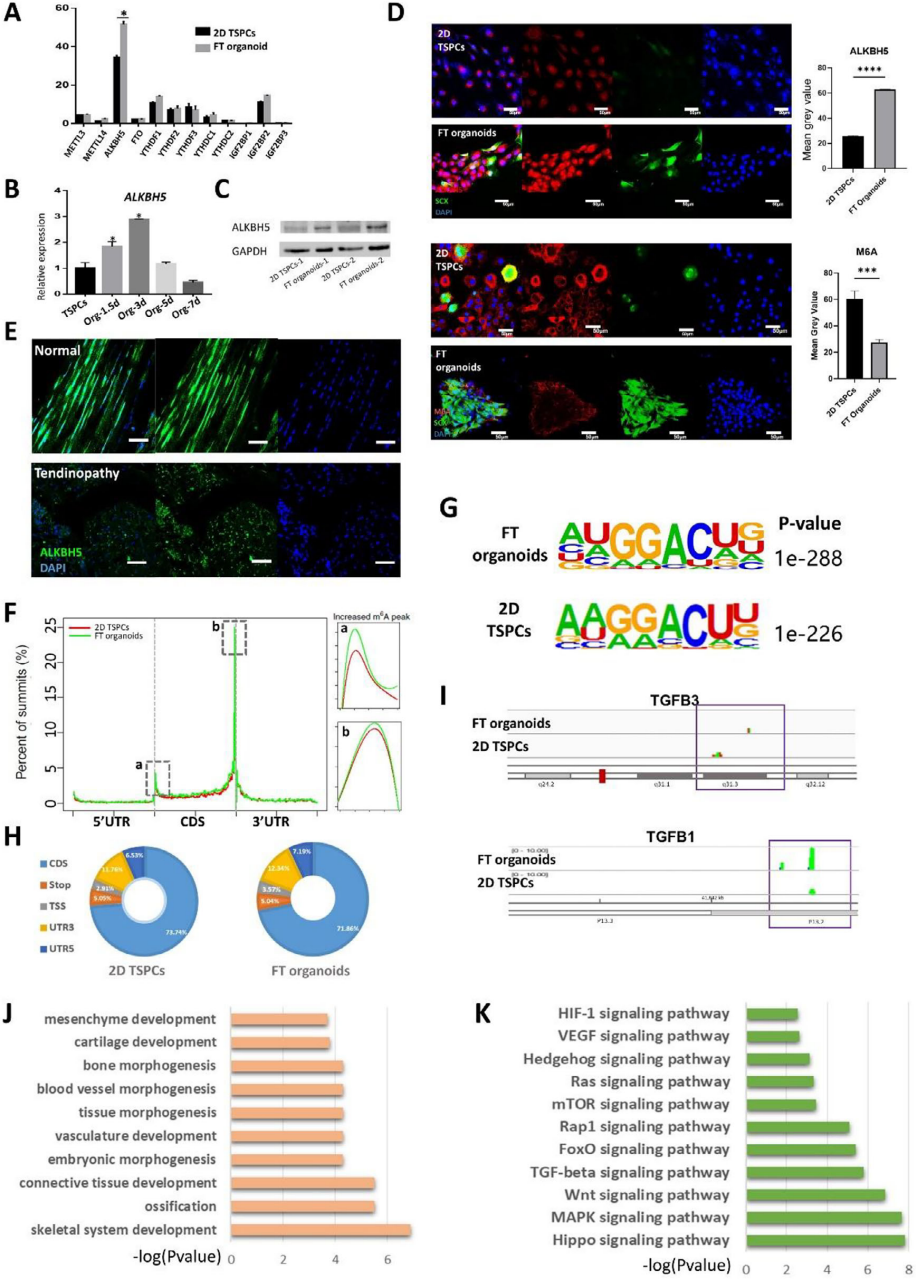
The demethylation Alkbh5 plays an important role in tendon development, and expressed higher in FT organoids than 2D TSPCs. (A) Expression of epigenetic enzymes in FT organoids and 2D TSPCs through the RNA‐seq data. (B) qRT‐ PCR of Alkbh5 in FT organoids and 2D TSPCs after culturing 1.5d, 3d, 5d, and 7d. (C) Western blot of ALKBH5 in in FT organoids and 2D TSPCs. (D) IF showed the protein expressions of ALKBH5 and its substrate m6A in FT organoids and 2D TSPCs. Nuclei were stained with DAPI. Mean gray value of ALKBH5 and m6A fluorescence intensity in FT organoids and 2D TSPCs. (E) IF showed the protein expressions of ALKBH5 in human normal tendon samples and tendinopathy samples. (F) Distribution of m6A peaks across the length of mRNA. (G) Top consensus motif identified by HOMER with m6A‐seq peaks of TSPCs in FT organoids and 2D TSPCs. (H) Graphs of m6A peak distribution showing the proportion of total m6A peaks in the indicated regions in FT organoids and 2D TSPCs. (I) The m6A peak patterns of tendon‐development associated genes by IGV viewer. (J) GO analysis of unique genes with m6A peaks in FT organoids. (K) GSEA analysis of unique genes with m6A peaks in FT organoids. ***, *p* < 0.001, ****, *p* < 0.0001 (Student's t test).

RNA methylation is recognized as an important epigenetic mechanism influencing stem cell pluripotency and differentiation [30, 31, 32]. Since 3D culture conditions can alter RNA methylation patterns [26], we investigated its potential role in maintaining organoid stemness and supporting tenogenic differentiation. We profiled the expression levels of mRNA methylases (*Mettl3, Mettl14*), mRNA demethylases (*FTO, ALKBH5*), m6A readers (*YTHDF1/2/3, YTHDC1/2*), and their regulator (*IGF2BP1/2/3*) in FT organoids from the RNA‐seq data. Interestingly, we observed a significant increase in the expression of ALKBH5 in FT organoids compared to other factors examine (**Figure** **5**A). qRT‐PCR results indicated that FT organoids significant increase the gene expression of *ALKBH5* from 1.5 days to 7 days during culturing process with the highest expression after 3 days of organoid formation (Figure 5B). Western blot and immunofluorescence staining also confirmed that the protein levels of ALKBH5 was upregulated in FT organoids with decreased m6A (Figure 5C,D). Furthermore, we confirmed the reduction of ALKBH5 expression in human tendinopathy by immunofluorescence (Figure 5E). These results indicated the pivotal role of ALKBH5 in orchestrating essential processes during tendon development and preserving its optimal functionality and overall health.

To investigate whether ALKBH5‐dependent m6A demethylation contributed to the differential gene expression patterns, we conducted a comprehensive m6A epitranscriptomic profiling comparing FT organoids with conventional 2D‐cultured TSPCs. We employed methylated RNA immunoprecipitation sequencing (MeRIP‐seq) to map the m6A methylomes of both FT organoids and 2D TSPCs, ensuring independent biological replicates for reliable analysis. In total, m6A‐seq identified 20 911 and 26 215 m6A peaks from 9856 to 10 171 m6A‐modified transcripts in FT organoids and 2D TSPCs, respectively (Figure S5A). The findings revealed that FT organoids exhibited higher m6A enrichment in the 5' untranslated region (UTR) and 3' UTR, indicating increased m6A modification in these regions. However, there was minimal impact on the coding sequence (CDS) region, suggesting that the m6A modifications predominantly occurred in the UTRs rather than the protein‐coding regions of the transcripts in FT organoids (Figure 5F). The GGACU motif was identified to be highly enriched within m6A sites in the FT organoids than in 2D TSPCs (Figure 5G). We further investigated the m6A distribution patterns within both total and unique peaks. A similar pattern of total and common m6A distribution in both groups was observed when the RNA species were divided into coding sequence (CDS), stop codon, transcription start site (TSS), 5’ UTR, and 3’ UTR regions of mRNAs (Figure 5H). We conducted a GO analysis on the unique genes identified in FT organoids and observed significant enrichment in multilineage differentiation functions. These findings were consistent with the results of the RNA‐seq GO analysis. Specifically, the enriched functions included connective tissue development, cartilage development, vasculature development, and skeletal system development (Figure 5J). Meanwhile, GSEA analysis of FT organoids unique target genes showed highly expression of musculoskeletal system development associated pathways, including Wnt, TGF‐β, MAPK and Hippo signaling pathways (Figure 5K). Results obtained from the IGV genome browser illustrated distinct m6A modifications on critical genes associated with the TGF‐β signaling pathway between FT organoids and 2D TSPCs (Figure 5I, FigureS5B). The substantial changes in mRNA methylation patterns between FT organoids and 2D TSPCs underscore the pivotal role of ALKBH5 in regulating mRNA methylation, potentially impacting the robust regeneration potential of FT organoids.

### 2.5 The Impact of ALKBH5 on the Maintenance of Stemness and Tenogenesis Within TSPCs‐derived FT Organoids, as Well as Its Role in Tendon Development

**FIGURE 6:**
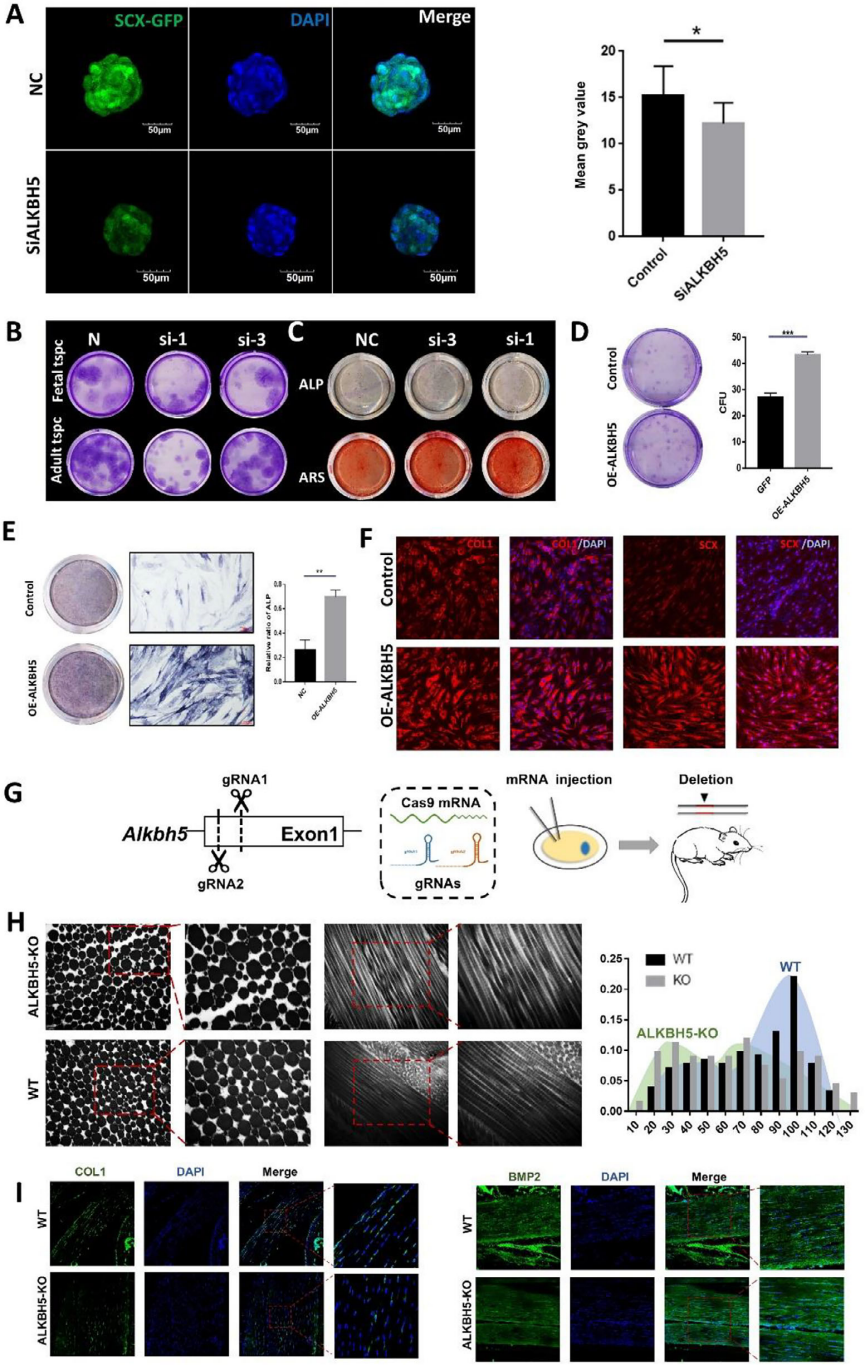
SiRNA silencing and overexpression of Alkbh5 influenced the stemness and tenogenesis of FT organoids. (A) Fluorescence intensity of scx‐GFP from siAlkbh5 and control scx‐GFP TSPCs. (B) CFU assay showing the self‐renewal of siAlkbh5 and control hTSPCs. Clones were visualized by methyl violet staining. (C) ALP staining (above) and ARS staining (below). (D) CFU assay showing the self‐renewal of Alkbh5‐overexpression and control hTSPCs. Clones were visualized by methyl violet staining. (E) ALP staining (left) and mean gray value (right) for mineralization. (F) Immunofluorescence of tendon‐associated genes of Alkbh5‐overexpression and control hTSPCs. (G) Schematic of the Alkbh5‐KO mice experimental protocol. (H) Transmission electron micrographs of cross‐sections of collagen fibrils in Alkbh5‐KO mice tendons and control groups. The number of large diameter collagen was more in the FT organoids than in the 2D group. (I) Immunofluorescence of tendon‐associated genes in ALKBH5‐KO mice tendons and control groups. All data are presented as the mean ± SD. *, *p* < 0.05. (Student's t test).

Subsequently, we conducted further investigations into the specific functions of Alkbh5 in FT organoids by employing siRNA targeting three different gene sites of Alkbh5. Following the silencing of Alkbh5 at sites 1 to 3 using the scx‐GFP mouse TSPCs system, a significant decrease in Scx expression was observed (Figures 6A, S5G). Western blot analysis further demonstrated a marked reduction in scleraxis (SCX) expression in siALKBH5 treated TSPCs (Figure S5G). CFU assays revealed that silencing *ALKBH5* site 1 or 3 markedly decreased the self‐renewal capacity of TSPCs (Figure 6B). Meanwhile, the competent osteogenic differentiation ability of TSPCs after silencing *ALKBH5* site 1 or 3 markedly was evidenced by diminishing ALP activity and less calcium mineralization (Figure 6C). The results unveiled that the silencing of *ALKBH5* resulted in a loss of phenotype, reduced clonality, and diminished osteogenic differentiation ability of TSPCs. Next, we transduced lentiviral constructs overexpressing ALKBH5 (OE‐ALKBH5), or gfp as a control (NC), into hTSPCs. CFU assays revealed that overexpression of *ALKBH5* significantly increased the self‐renewal capacity of TSPCs (Figure 6D). The osteogenic differentiation ability of TSPCs was assessed through ALP assays, revealing that the overexpression of Alkbh5 significantly enhanced TSPCs' osteogenesis (Figure 6E).

To investigate the role of ALKBH5 in tendon development in mice, a targeted deletion of *Alkbh5* in mice was created by deleting a part of exon 1 of the Alkbh5 gene using CRISPR‐Cas9 technology [33] (Figure 6G, Figure S5E). To investigate the changes in collagen fibrils, we conducted an analysis of TEM images of the mice Achilles tendons (Figure 6H). Statistical analysis revealed that Alkbh5‐KO mice exhibited a higher abundance of fine collagen compared to control mice. Immunofluorescence staining showed that the expressions of tendon‐related proteins SCX, COL1, BMP2, NES and TNMD were decreased in tendon tissues of Alkbh5‐KO mice (Figure 6I). Consistently, western blotting showed a decrease in SCX protein levels in ALKBH5‐KO mice (Figure S5E). The findings highlight the significance of ALKBH5‐mediated processes, including mRNA demethylation and gene expression regulation, in orchestrating the intricate molecular mechanisms underlying tendon cell phenotype maintenance and the intricate processes involved in tendon development.

### 2.6 Single‐cell Level Reveals the Role of ALKBH5+ TSPCs in FT Organoids

**FIGURE 7:**
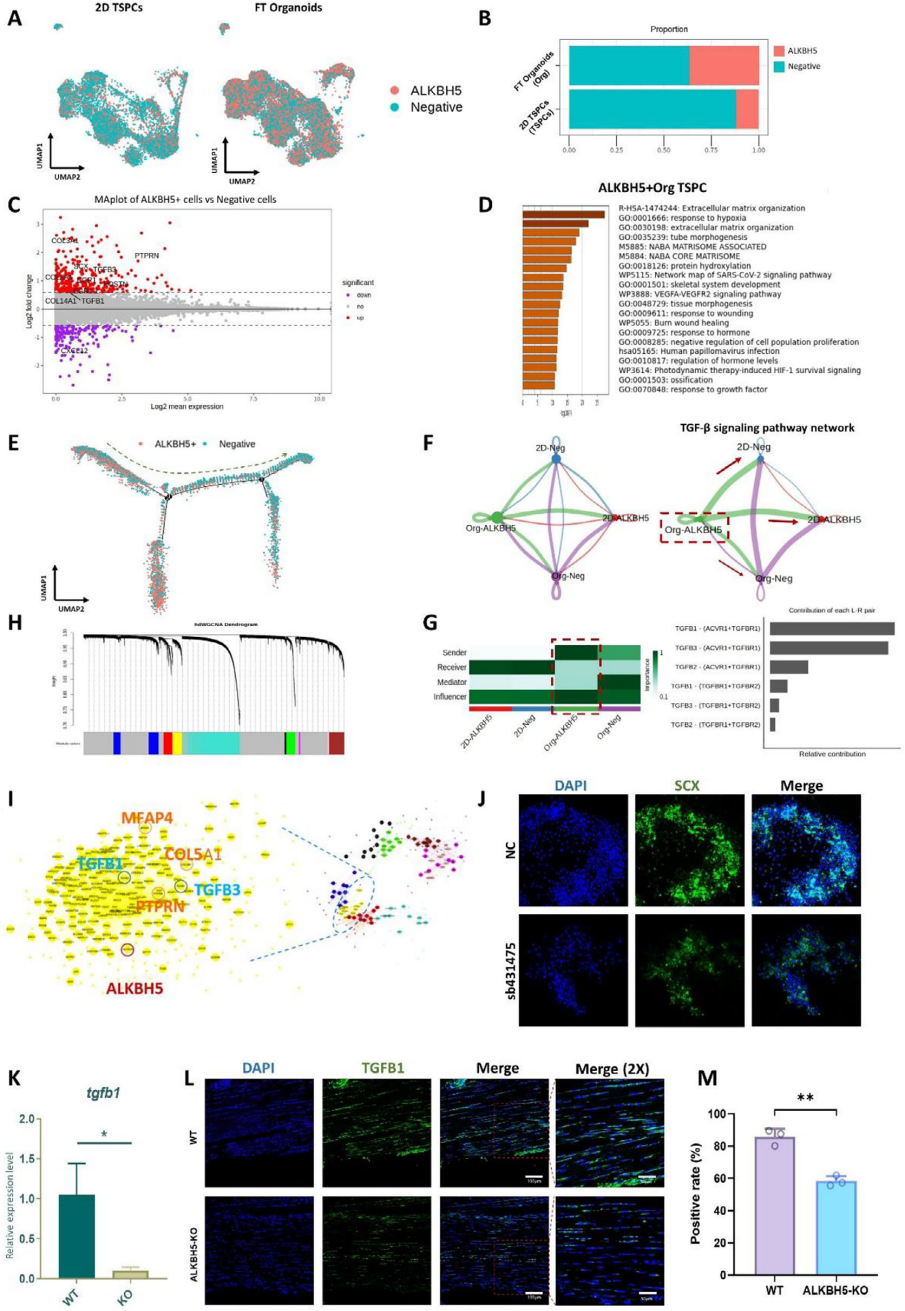
ALKBH5 regulated tenogenesis phenotypes through TGFβ signaling pathway in FT organoids by scRNA‐seq analysis. (A) UMAP visualization of single cells from FT organoid, where individual points correspond to single cells. Cells are colored by ALKBH5+ and ALKBH5‐ TSPCs. (B) Cell type ratios for the ALKBH5+ and ALKBH5‐ TSPCs in FT organoids and 2D TSPCs. (C) Volcano plot of differential genes in the ALKBH5+ and ALKBH5‐ TSPCs. (D) Bar plot of GO analysis of differential genes in the ALKBH5+ TSPCs. (E) Pseudo‐temporal trajectory of cells from FT organoids and 2D TSPCs combined by Monocle. The color represented ALKBH5+ and ALKBH5‐ TSPCs. (F) Cell‐cell interaction among ALKBH5+ FT organoids, ALKBH5‐ FT organoids, ALKBH5+ 2D TSPCs, and ALKBH5‐ 2D TSPCs through Cellchat, and TGFB signaling pathway among ALKBH5+ FT organoids, ALKBH5‐ FT organoids, ALKBH5+ 2D TSPCs, and ALKBH5‐ 2D TSPCs through Cellchat. (G) senders and receptors in TGFβsignaling pathway through Cellchat. (H) hdWGCNA dendrogram of weighted gene co‐expression network in FT organoids and 2D TSPCs combined. (I) hdWGCNA co‐expression networks of yellow module. (J) Immunofluorescence of Scx expressed in TGF‐β signaling pathway inhibitor (sb431475) cultured TSPCs and control. (K) qPCR of TGFB1 in ALKBH5‐KO mice tendons and control. (L) Immunofluorescence of TGFB1 expression in ALKBH5‐KO mice tendons and control. (M) Comparison of the positive rates of TGFB1 in the tendons of ALKBH5‐KO mice and WT mice. All data are presented as the mean ± SD. *, *p* < 0.05; **, *p* < 0.01. (Student's t test).

To gain a deeper understanding of how ALKBH5 influences the cellular behaviors, including stemness maintenance and tenogenesis, we detected ALKBH5+ cells (ALKBH5 counts ≥1) in FT organoids and 2D TSPCs in scRNA‐seq data. We found a higher proportion of ALKBH5+ cells in the FT organoids compared to the 2D TSPCs (**Figure** **7**A,B). We compared the differential genes between ALKBH5+ cells and negative cells (ALKBH5 counts = 0) and found that tendon‐related genes *COL1A1, COL14A1, SCX, COL3A1, PTPRN, POSTN, TGFB1, TGFB3* were elevated in ALKBH5+TSPCs (Figure 7C). The GO analysis of differentially expressed genes in ALKBH5+ TSPCs revealed their predominant involvement in various biological processes, including hypoxia response, extracellular matrix organization, HIF‐1 survival signaling, response to wounding, and ossification (Figure 7D). These findings suggest that ALKBH5+ TSPCs are specifically associated with cellular responses to hypoxic conditions, remodeling of the extracellular matrix, activation of survival pathways mediated by HIF‐1. Through pseudo‐temporal analysis of all cells using Monocle, we observed that ALKBH5+ TSPCs were mainly situated in the early portion of the pseudo‐temporal trajectory, while ALKBH5‐negative TSPCs were primarily distributed in the middle and late stages of the trajectory (Figure 7E). These results indicate that the high expression of ALKBH5 is a crucial factor in maintaining the fetal‐like characteristics of FT organoids. To investigate whether ALKBH5+ cells exert regulatory effects on negative cells through intercellular interactions, we utilized CellphoneDB [34]—a public repository of curated ligands, receptors, and their interactions used to analyze cell‐cell communication from single‐cell transcriptomics data—for systematic cell‐to‐cell interaction analysis. The analysis results provided an indication that ALKBH5+ TSPCs may act as donor cells, secreting factors associated with the TGF‐β signaling pathway, including TGFB1, TGFB2, and TGFB3, to the negative cells (Figure S5C). Cellchat [35] also used to analyze the interaction among FT organoid ALKBH5+ TSPCs (Org‐ALKBH5+), FT organoid ALKBH5 negative TSPCs (Org‐Neg), 2D ALKBH5+ TSPCs (2D‐ALKBH5+) and 2D organoid negative TSPCs (2D‐Neg) (Figure 7F). The analysis results suggested that Org‐ALKBH5+ TSPCs were the main sender of TGF‐β signaling pathway, while 2D‐ALKBH5‐TSPCs and 2D‐ALKBH5+ TSPCs were the main receiver (Figure 7G). These findings suggest that FT organoid‐ALKBH5+ TSPCs primarily exert regulatory effects on neighboring cells through the TGF‐β signaling pathway. To validate this phenomenon, we investigated the expression of TGFB1 in ALKBH5+ and negative TSPCs within both FT organoids and 2D groups. Interestingly, our results demonstrated that Org‐ALKBH5+ TSPCs exhibited the highest expression of TGFB1, which was consistent with the predicted outcomes (Figure S5D). The knockdown of ALKBH5 with siRNA or ALKBH5 knockout both led to reduced expression levels of TGFβ1 and SCX in TSPCs (Figure S5E,G). We also employed hdWGCNA [36] to analyze the co‐expression network of gene expressions in FT organoids. This method involves decomposing highly similar cells into cell modules and conducting network analysis, which helps us understand the co‐expression relationships between ALKBH5 and other genes in FT organoids (Figure 7H). The yellow module is a representative module of FT organoids and ALKBH5+ cells gene expression (most expressed in FT organoid group and almost not expressed in 2D group) (Figure 7H,I). The yellow module GO terms included connective tissue development, extracellular matrix organization, response to hypoxia, and signaling by TGF‐β receptor complex (Figure S5F). The expression of yellow module was the highest in ALKBH5+ TSPCs (Figure S5I). This indicated that FT organoids and ALKBH5+ TSPCs share similar gene co‐expression patterns, again validating the major regulatory role of ALKBH5+ in FT organoids. The yellow module showed the co‐expression of ALKBH5 with the key genes of TGF‐β signaling pathway (TGFB1 and TGFB3) and tendon‐associates genes (COL5A1 and PTPRN) (Figure 7I). Therefore, we hypothesized that TGF‐β signaling, as a downstream target of ALKBH5, receives ALKBH5 demethylation to regulate tendon phenotype of TSPCs. The differential mRNA methylation sites in key genes associated with the TGF‐β signaling pathway observed in the MeRIP‐seq results further support this notion (Figure 5I, Figure S5B). Inhibition of TGF‐β signaling pathway by TGF‐β signaling pathway inhibitor sb431475 resulted in decreased expression of SCX in the FT organoids in vitro (Figure 7J, Figure S5H). The regulation of Tgfb1 by Alkbh5 in mice has also been verified. We observed that Tgfb1 expression in TSPCs of Alkbh5‐KO mice was also significantly decreased compared with WT mice (Figure 7K). Immunofluorescence staining also demonstrated a significant decrease in the proportion of TGFB1‐positive cells in the tendons of Alkbh5‐knockout (KO) mice (Figure 7L,M). These findings suggest that ALKBH5 plays a role in regulating the tendon phenotype by demethylating key genes involved in the TGF‐β signaling pathway.

## 3 Discussion

In this study, we generated organoids from TSPCs, which outperformed conventional 2D cultured TSPCs in terms of their ability to restore the phenotypic characteristics and tenogenic potential of TSPCs in vitro. Moreover, these FT organoids demonstrated the capability to promote the regeneration of tendon collagen fibers in vivo. ALKBH5 exerted control over the transcription of key stem cell genes and TGF‐β signaling pathways, highlighting its pivotal role in guiding the regeneration process, representing a promising and advanced strategy for tendon regeneration.

Currently, numerous studies have utilized organoids in tissue engineering approaches for tissue repair, including brain organoids, cardiac organoids, kidney organoids [37, 38, 39, 40]. Related, MSCs combined with biological scaffolds have been used to construct organoids such as bone [41], cartilage, epithelial [42], vascular tissue and heart tissue. The field of tendon therapy currently lacks stable and effective organoids for tissue engineering. Our study addresses this by developing FT organoids that emulate a fetal‐like tendon state with robust stem cell properties. These organoids demonstrate remarkable regenerative potential in adult tendon repair, showing significant therapeutic efficacy despite the inherently limited regenerative capacity of mature tendon tissue. Our tendon organoids differed from conventionally recognized tendons that possess physiological mechanical tension. Due to the suspension culture method, the organoids did not develop the characteristic dense, parallel fibrous structure. This unique characteristic rendered them more suitable as seed organoids for tendon tissue engineering, as they allowed for the study of TSPCs in a state closer to their in vivo condition by decoupling the dominant mechanical effects. This state potentially enabled them to exert enhanced regenerative functions during tissue repair. Future work could build upon this foundation by incorporating mechanobiological components and applying mechanical stimulation in vitro to achieve specific research objectives. In addition, tendons are suitable for allogeneic transplantation, which supports the feasibility of using our tendon organoids in an allogeneic transplantation context. Based on our animal studies and existing clinical evidence from cell therapies, our organoid approach, if translated to clinical practice, could potentially offer a substantially reduced cell dosage requirement and decreased overall treatment costs. The long‐term benefits of FT organoid therapy will also be a focus of future research.

Recent studies have revealed that 3D culture architectures can modulate epigenetic modifications, enabling pluripotent stem cells to revert to a naive state [43]. This compelling evidence suggested that the suspended 3D microenvironment of organoids may serve as a fundamental determinant of mRNA epigenetic remodeling. The mRNA m6A modification has been reported to modulate the specification in multiple stem or progenitor cells, including haematopoietic stem cells, neural stem cells, glioblastoma stem cells and MSCs [6, 26, 27, 33, 44, 45]. M6A functions as a modulator or balancer in pluripotent or multipotent stem cells for cell fate determination. Therefore, exploring the mechanisms of mRNA methylation modification in stem cells, and purposefully manipulating them, will become an important direction in the in vitro cultivation of stem cells or organoids.

ALKBH5 belongs to the AlkB family of nonheme Fe (II)/α‐ketoglutarate‐dependent dioxygenases, whose activity is iron‐dependent. Our study first explored the role of mRNA demethylase ALKBH5 in the maintenance of tendon stem cell phenotype in FT organoids microenvironment and in tendon regeneration. In previous studies, ALKBH5 has been reported to be involved in regulating the m6A modification pattern under hypoxia stress, thereby affecting cell proliferation, stem cell characteristics and tumor development [30]. In our study, ALKBH5+ TSPCs were found to regulate the hypoxia pathway, which may be responsible for the increased expression of ALKBH5 in FT organoids. ALKBH5 has also been previously reported to regulate TGF‐β/SMAD signaling in non‐small cell lung cancer [45]. In combination with our results, this indicates that the TGF‐β signaling pathway and ALKBH5 may have more intricate mutual regulatory interactions. Our findings imply that ALKBH5 regulation may also serve as a potential driver of TGF‐β expression changes in bone, cartilage, and other tissues, offering a new direction for future research on this pathway.

This study aimed to develop TSPCs‐derived FT organoids as highly promising seed cells for tendon repair and regeneration in tissue engineering, which provided a novel and effective strategy for tendon repair and regeneration, offering new insights into the development of advanced tissue engineering approaches and bringing us closer to clinical applications in the field of orthopedic medicine.

## 4 Experimental Section

### 4.1 The Isolation, Culturing of TSPCs, and Generation of FT Organoids

The TSPCs were isolated and cultured following established protocols [47]. The acquisition, isolation, and culture procedures were conducted in compliance with the guidelines set forth by the Ethics Committee of the Second Affiliated Hospital, School of Medicine, Zhejiang University (Approval No. 12019001166), adhering to the principles of the 1964 Helsinki Declaration and its subsequent amendments, or other comparable ethical standards. The animal study protocol was approved by the Zhejiang University Institutional Animal Care and Use Committee (ZJU20200049). Human tendon samples were obtained from surgically resected, damaged or pathological tendon tissues during orthopedic or tendon repair procedures, primarily including the rotator cuff and Achilles tendon. Normal TSPCs were isolated from patients aged 20 to 40 years, while aged TSPCs were derived from patients over 60 years old.

Mouse tail tendon stem/progenitor cells (TSPCs) were isolated from the tail tendon of ScxGFP transgenic mice as described previously [48]. The ScxGFP transgenic mice were kindly provided by Dr. Ronen Schweitzer (Oregon Health & Science University, Oregon, USA). Briefly, tissue samples were dissected into small fragments and enzymatically digested using 0.20% type 1 collagenase in low‐glucose Dulbecco's modified eagle medium (L‐DMEM) for 1 h at 37°C. The resulting single‐cell suspensions were then cultured in a growth medium comprising L‐DMEM supplemented with 10.00% fetal bovine serum (FBS) and 1% penicillin‐streptomycin solution (PS) for 8–10 days at 5% CO2 and 37°C. Upon reaching 80–90% confluence, hTSPCs were passaged for use in the present study.

For FT organoids culturing, approximately 1 × 10^5^ TSPCs were digested, collected, and resuspended in a specialized serum‐free medium for FT organoids. The medium formulation was as follows: DMEM/F12 base supplemented with N2 supplement (GIBCO, 17502001), B27 supplement (GIBCO, 17504044), 10 ng/mL basic fibroblast growth factor (bFGF, Peprotech, AF‐100‐18B), and 20 ng/mL epidermal growth factor (EGF, peprotech, AF‐100‐15). The cell suspension was then transferred to an ultra‐low attachment 6‐well plate and maintained at 37°C with 0.5% CO_2_. According to these protocols, the organoids will be able to be replicated in other laboratories.

### 4.2 Colony‐Forming Unit Assay

Colony‐forming unit (CFU) assays were used to characterize the self‐renewal potential of cells as previously described [4]. The colonies formed were stained with 1% methyl violet (Sigma) and then washed in phosphate‐buffered saline.

### 4.3 Assessment of Multipotent Differentiation Capacity

The multi‐potential assays of hTSPCs, including osteogenesis, adipogenesis, and chondrogenesis, were conducted following established protocols [49]. For osteogenic differentiation, hTSPCs were cultured in high glucose‐DMEM (H‐DMEM) supplemented with dexamethasone (Sigma‐Aldrich) for 7 days. Osteogenic differentiation was confirmed by alkaline phosphatase staining (ALP, Beyotime) and Alizarin Red S (ARS) staining. Adipogenesis was induced by culturing the cells in MesenCultTM adipogenic differentiation kit (Human) (StemCell Technologies) and by Oil Red O staining.

### 4.4 Assessment of Tenogenesis

As described previously [3], upon reaching confluence, hTSPCs from FT organoids and 2D culturing systems were cultured in DMEM supplemented with 10% (vol/vol) fetal bovine serum and 50 mg/mL ascorbic acid (A8960, Sigma). A multilayered cell sheet was detached from the substratum by applying a small roll‐up force after 14 days in culture.

### 4.5 Immunofluorescence

Cells and samples were fixed and processed by standard histological procedures as described previously [4]. The samples were incubated with primary antibodies overnight at 4°C. The primary antibodies were utilized anti‐COL1 (Proteintech, 14695‐1‐AP), anti‐COL14 (Abcam, ab58084), anti‐TNMD (Abcam, ab81328), anti‐SCX (Abcam, ab58655), anti‐BMP2 (Beyotime, AF0075), anti‐IL1β (Abcam, ab9722), anti‐EGR1 (Proteintech, 22008‐1‐AP), anti‐RUNX2 (Abcam, ab76956), anti‐EYA2 (Sigma, HPA027024), anti‐Tnmd (Abcam, ab81328), anti‐MKX (Lifespan, LS‐B8063‐50), anti‐m6A (Abcam, ab190886), anti‐ALKBH5 (Sigma, HPA007196), anti‐TGFβ1 (Santa Cruz, sc‐130348), anti‐Tenascin‐C (Santa Cruz, sc‐130348), and anti‐Lamin A+C (Abcam, ab108595). The goat anti‐rabbit or mouse second antibody were conjugated with Alexa Fluor 488 or 546 fluorescent dye (Invitrogen). Cell nuclei were stained with DAPl. Fluorescent images were obtained by a confocal microscope (Olympus). The Olympus confocal microscope (Model BX61) used in this study was equipped with visible light lasers at wavelengths of 405 nm, 488 nm, 561 nm, and 635 nm. For this study, the excitation wavelengths were set as follows: 488 nm for green fluorescence, 561 nm for red fluorescence, and 405 nm for DAPI fluorescence.

### 4.6 Transmission Electron Microscopy

For transmission electron microscopy imaging to assess the collagen fibril diameter and fiber alignment, cell‐sheets and tissue specimens were fixed and processed by standard procedures as described previously [35]. At least 200 collagen fibrils from at least three independent cell sheets and _500 collagen fibrils from at least three independent tissue samples were measured and analyzed at least three times. Image‐Pro Plus (IPP 6.0, Media Cybernetics, Rockville, MD, http://www.mediacy.com.cn/cn/index/index.asp) software was used to measure the collagen fibril diameters.

### 4.7 RNA Isolation, Reverse Transcription, and qRT‐PCR

The mRNA levels of tenogenesis‐specific genes were assessed using real‐time PCR, as previously described [35]. All primer sequences (Invitrogen) were designed using Primer 5.0 software and are summarized in Supporting Information Table S1. Representative results are shown as target gene expression normalized to the reference housekeeping gene *Gapdh*/*GAPDH*. For mRNA expression in TSPCs [34], RNA samples were prepared from at least three independent samples and analyzed at least three times.

### 4.8 Patellar Tendon Injury and Repair Animal Model and Alkbh5‐KO Mouse Model

A rat model of patellar tendon injury was used in this study. The Institutional Animal Care and Use Committee of Zhejiang University approved the experimental protocol (ZJU20200049). Twenty‐four skeletally mature Sprague‐Dawley rats weighing 200–250 g were used for the rat model of patellar tendon injury (with 48 hind limbs). Both male and female rats were used for the experiments. The rats were treated with cyclophosphamide (150 mg/kg) 24 h before the operation. To create the tendon defect, the central one‐third of the patellar tendon (∼1 mm in width) was removed from the distal apex of the patellar to the insertion of the tibia tuberosity, according to a well‐established protocol in our previous work [36]. Doxycycline (2 mg/mL; Biosharp, HEFEI, China, http://www.biosharp.cn) was administered in drinking water with 10 mg/mL sucrose (Sinopharm Chemical Reagent, Co., Ltd, Shanghai, China, http://www.sinoreagent.com/). 2 days before in situ repair, 2D TSPCs were cultured in FBS‐free media for 24 h. 1 × 10^5^ cells were generated into FT organoids for each implantation.

In the experimental limbs, tendon defects were treated with FT organoids of 1 × 10^5^ TSPCs and 2D TSPCs with equal quantity in 20 µL fibrin gel, whereas the control limbs were treated with 20 µL fibrin gel alone. 1, 2, and 4 weeks after implantation, the repaired patellar tendons were processed for histological analysis and hydroxyproline assay.

The Alkbh5 (Embryonic knockout (KO)/B6 mouse model was created as previously description [33]. The Alkbh5‐KO mouse model with a C57BL/6 background was generated. To disrupt early exon 1 of the Alkbh5 gene, two guide RNAs (gRNAs) were designed. The selection of gRNAs was based on off‐target scores obtained from http://www.genome‐engineering.org. By employing this approach, the specific disruption of early exon 1 of the Alkbh5 gene was achieved in the mouse model, enabling the investigation of the resulting phenotypic changes and the study of Alkbh5 function in development. Twelve mice (ALKBH5‐KO and controls, 8‐week‐old, 18–20 g) were used, with equal numbers of males and females (n = 6 per sex).

All experimental animals were housed at the Zhejiang University Laboratory Animal Center following standard protocols.

### 4.9 Histologic Evaluation

Following 24–28 h of fixation in 4% paraformaldehyde, the tissue samples were dehydrated through a graded ethanol series and embedded in paraffin. Consecutive 7 µm sections were prepared using a Leica microtome. Hematoxylin and eosin (H&E) staining and histological evaluation were performed according to established methods [10]. A blinded semi‐quantitative scoring system was implemented by an independent evaluator, assessing six key parameters: fiber structure, fiber alignment, nuclear morphology, inflammatory cell infiltration, vascular density, and cellularity in the repaired tendon region. Each parameter was scored on a 0–3 scale, with 0 representing normal tissue and 3 indicating severe abnormality. Two random sections per sample—one spanning the normal‐repaired tissue interface and one from the central repair area—were selected for assessment. Furthermore, collagen fiber organization was evaluated using Masson's trichrome and Sirius Red staining, while collagen maturity was examined under polarized light microscopy.

### 4.10 Single Cell Capturing and scRNA‐Seq Data Analysis

FT organoids and 2D TSPCs were digested with 0.25% collagenase as single cell. Single cell capture, cDNA library preparation and sequencing were performed at Singleron Biotechnologies (Nanjing, Jiangsu, China). Raw sequencing reads were processed with Perl scripts to ensure the quality of data used in further analysis. We removed the adaptor‐polluted reads (reads containing more than 5 adapter‐polluted bases) and the low‐quality reads (reads with the number of Quality value less than 19 accounting for more than 15% of total bases). Reads with number of N bases accounting for more than 5% were also discarded. After obtaining the digital gene expression data matrix, we used Seurat [50] (V2.3.4) for dimension reduction, clustering and differential gene expression analysis. For quality control, we excluded cells in which expression of less than 2000 or more than 6000 genes were detected. Genes that were ascertained in more than 2 cells were kept. To reduce the dimensionality of the data, a dimensional reduction technique was applied, and cell clusters were subsequently identified based on the most significant principal components. The identification of genes significantly enriched in each cell cluster was carried out using the default algorithm implemented in Seurat. To gain insights into the functional characteristics of these cell clusters, the resulting marker gene lists were subjected to functional annotation analysis relative to Gene Ontology terms. This analysis was conducted using Metascape, a tool specifically designed for functional annotation and analysis of gene lists [51]. Ligand‐Receptor was analyzed by CellphoneDB [34]. The cell‐cell interaction was analyzed by CellChat [52]. The trajectory analysis was performed using Monocle (v2.10.1) [53].

### 4.11 RNA‐Seq Experiments and Data Analysis

The total RNA was extracted from tissue samples with Trizol reagent (TAKARA). cDNA was synthesized using SuperScript II reverse transcriptase (Invitrogen), followed by second‐strand cDNA generation with the NEBNext mRNA Second Strand Synthesis Module (NEB). Libraries were prepared using the Nextera XT kit (Illumina) and sequenced on an Illumina X‑Ten platform. Reads were aligned to the hg38 reference genome via bowtie2 (default parameters) All the statistical analyses were conducted using R statistical programming languages. Differential gene expression analysis was performed using DESeq2. In our analyses, a gene was considered to be expressed in a sample if its count value was equal to or greater than 1 in the sample. Genes with count values of zero across all samples were removed. DEGs were defined as fold change ≥2 and p value ≤ 0.05. Gene Ontology and KEGG analysis was performed using Metascape.

### 4.12 SiRNA and Lentiviral Infection

The human ALKBH5 gene (NM_177595.4) were synthetized by JiKai, Co. (Shanghai, China, http://genechem.bioon.com.cn/). Egfp‐FUW tetracycline‐inducible lentiviral vector was used as the control. For the ALKBH5 overexpression studies, human TSPCs were infected with lentiviral vectors carrying human ALKBH5. For lentiviral infection, cells at 50% confluence were infected in the presence of 10 ng/mL polybrene (28728‐55‐4, Sigma, Saint Louis, MO, http://www.sigmaaldrich. com/china‐mainland.html). After infection, the cells were treated with 4 mg/mL doxycycline (Sigma) in the medium to activate tetracycline‐inducible gene expression. The cells were selected with Zeocin (Invitrogen) at 200 mg/mL for 10 days. Transduced cells at passages 3 and 5 after selection were used for functional studies. Small interfering RNAs (siRNAs) targeting ALKBH5 were directly synthesized (Ribo, China). Transfection of plasmids or siRNAs was conducted using Lipofectamine 2000 (Beyotime, China). Cells were collected for further experiments 2 days after transfection.

### 4.13 Methylated RNA Immunoprecipitation Sequencing

MeRIP Methylated RNA Immunoprecipitation was performed following previously established protocols [54]. In brief, RNA was extracted and purified was fragmented and denatured. These fragments were incubated with an anti‐m6A antibody and protein A/G magnetic beads in an immunoprecipitation buffer at 4°C overnight. The antibody‐bound methylated RNA was subsequently eluted using m6A, and the eluted RNA was further purified for subsequent MeRIP sequencing. The MeRIP sequencing was conducted by Origingene (Shanghai, China).

### 4.14 MeRIP‐Seq Data Analysis

Sequencing was performed using the Illumina HiSeq 4000 platform, following the manufacturer's instructions, with single‐end reads of 50‐bp length. The generated reads were aligned to the human genome version GRCh38 using the STAR alignment tool.

### 4.15 Statistical Analysis

The quantitative data were presented using the mean ± standard deviation (SD). When variables presented normal distribution and equal variance, student's t‐test was performed for comparison of data of paired samples and one‐way analysis of variance (ANOVA) was used for multiple group comparisons. Statistical analysis was performed using Microsoft Excel. The significance level is presented as * *p* < 0.05, **p < 0.01, ***p < 0.001, or ****p < 0.0001. Values of *p* < 0.05 were considered to be statistically significant.

## Author Contributions

Tian Qin designed the study, performed data curation, conducted the principal experiments analysis, visualization, and wrote the manuscript. Aini Pan and Zhuoning Miao contributed significantly to in vivo and in vitro validation experiments and data visualization. Yanyan Zhao, Xianan Mo, and Junyu Guo participated in experimental validation and data visualization. Heng Sun and Qiangqiang Zheng assisted in manuscript editing. Chunmei Fan supported data collection and visualization. Bingbing Wu analyzed single‐cell RNA sequencing data. Weiliang Shen aided in data curation and funding acquisition. Jun Lu and Xi Jiang provided the genetically modified mice, and validation support. Zi Yin and Xiao Chen jointly supervised the study, secured funding, and guided the project design and writing.

## Funding

This work was supported by the National key research and development program of China (2022YFA1106800), NSFC grants (T2121004, 82222044, 32471211), Key R&D Program of Zhejiang (2024SSYS0026), Natural Science Foundation of Jiangsu Province (BK20250360), Dr. Li Dak Sum & Yip Yio chin Development Fund for Regenerative Medicine, Zhejiang University.

## Conflicts of Interest

The authors declare no conflict of interest.

## Supporting information




**Supporting File 1**: advs73853‐sup‐0001‐SuppMat.docx.

**Supporting File 2**: advs73853‐sup‐0002‐TableS1.xlsx.

## Data Availability

The data that support the findings of this study are openly available in The China National Center for Bioinformation at https://ngdc.cncb.ac.cn/gsub/, reference number 7520.
