# Correction to “Tendon Organoids Enable Functional Tendon Rejuvenation Through ALKBH5‐Dependent RNA Demethylation”

**DOI:** 10.1002/advs.75680

**Published:** 2026-05-14

Tian Qin, Aini Pan, Zhuoning Miao, Yanyan Zhao, Xianan Mo, Heng Sun, Chunmei Fan, Junyu Guo, Bingbing Wu, Weiliang Shen, Qiangqiang Zheng, Jun Lu, Xi Jiang, Zi Yin, Xiao Chen, “Tendon Organoids Enable Functional Tendon Rejuvenation Through ALKBH5‐Dependent RNA Demethylation,” *Advanced Sci*ence 13, no. 17 (2026): e08376, https://doi.org/10.1002/advs.202508376.

**Error Description**:

The same BMP2 immunofluorescence image was unintentionally used in Figures S1D and S3C. This duplication occurred because the two panels correspond to the same experimental group, leading to our accidental reuse of the identical image during figure assembly.

In Figure S1D, this image was intended to show BMP2 staining as one of the markers for different cell clusters in FT organoids (single‐cell RNA‐seq analysis results validation).

In Figure S3C, the same image was aimed to compare BMP2 expression between FT organoids and 2D TSPCs.

**Correction**:

The corrected Figure S1D (containing the proper BMP2 immunofluorescence image from the intended experimental condition) has been replaced in the online version of the article. Figure S3C remains unchanged. The corrected figure now accurately represents the intended experimental data for the validation of the markers for different cell clusters.

**Impact**:

We have carefully re‐evaluated the paper and confirm that this error does not affect the overall conclusions of the study. Moreover, the figure revision will not alter the framework, data logic, or core findings of the original manuscript.

We apologize for this error.

## Supporting information




**Supporting file**: advs73853‐sup‐0001‐SuppMat.docx

