# Supplementary material for: Correction to “Tendon Organoids Enable Functional Tendon Rejuvenation Through ALKBH5‐Dependent RNA Demethylation”
Source: Adv Sci (Weinh). 2026 May 14;13(34):e75680. doi: 10.1002/advs.75680 (PMC13285170; doi:10.1002/advs.75680)
Supplement: Supplementary file 1 — Supporting file: advs73853‐sup‐0001‐SuppMat.docx [file ADVS-13-e75680-s001.docx]

**Supplementary** **Figures**


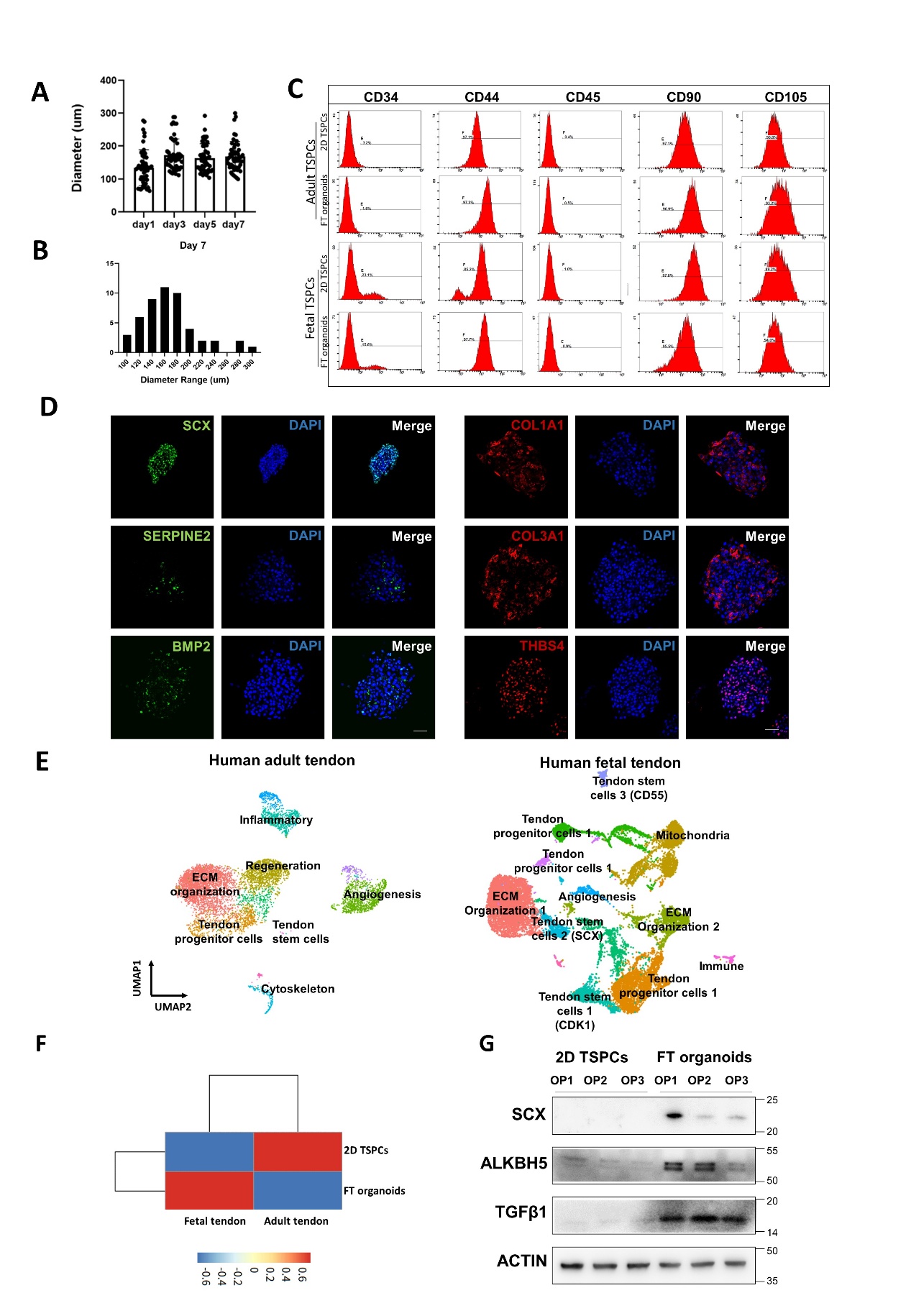


**Fig. S1. Characterization of the FT organoids.**

(A) Diameter quantification of FT organoids cultured for 1-7 days. (B) Size distribution of FT organoids following 7 days in culture. (C) Flow cytometry analysis revealed CD34, CD44, CD45, CD90, CD105 in adult and fetal hTSPCs. (D) Immunofluorescence staining validated the marker genes of each cluster. (E) UMAP visualization of single cells from fetal and adult tendons, where individual points correspond to single cells. Cells are colored by annotated clusters. (F) Correlation between FT organoids and 2D TSPCs and fetal/adult tendons. The colors from blue to red represented the correlation levels from low to high. (G) Protein expression of SCX, ALKBH5, and TGFβ1 were detected using tendon cells from three older patients (over 60 years old, designated OP1, OP2, OP3) by Western blot.


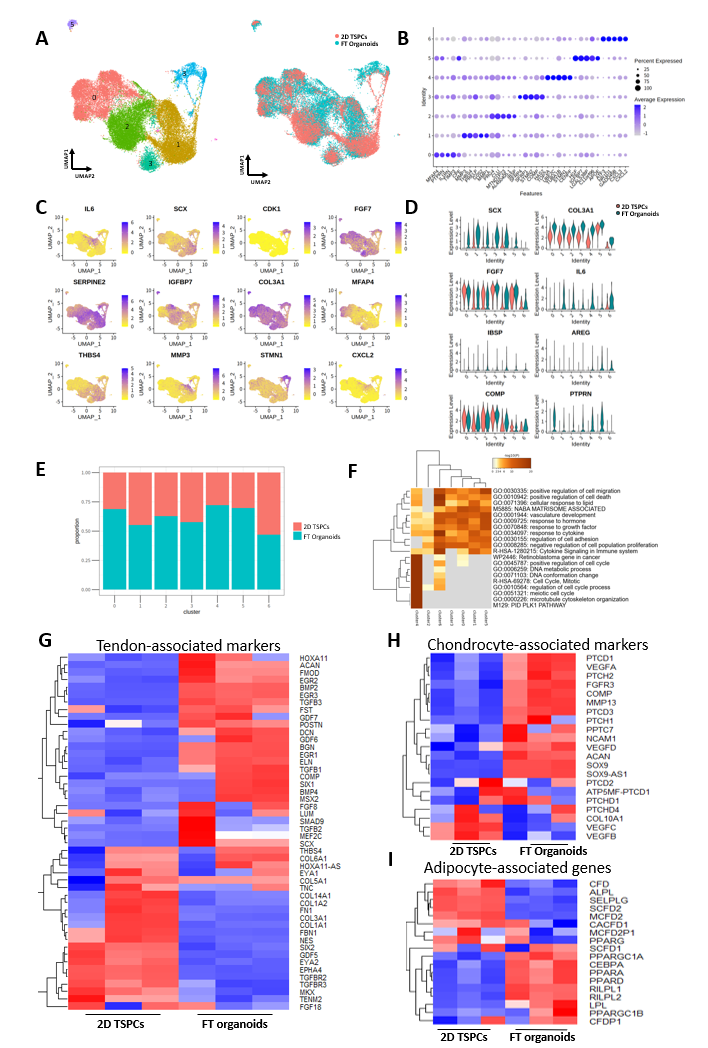


**Fig. S2. ScRNA-seq analysis of TSPCs from FT organoids and 2D TSPCs combined.**

(A) UMAP visualization of single cells from FT organoid and 2D TSPCs c, where individual points correspond to single cells. Cells are colored by annotated clusters (left) and FT organoids and 2D TSPCs (right). (B) Dot plot of marker genes of each cluster. (C) Expression patterns of well-known tendon-associated markers were projected onto the UMAP. The colors from yellow to purple represented expression levels from low to high. (D) Violin plots of well-known tendon-associated markers of each cluster splited by groups. (E) Cell type ratios for the FT organoids and 2D TSPCs. The colors represent different cell types. (F) Heatmap of GO analysis of differentiated genes of each cluster. The colors from yellow to dark red represented expression levels from low to high. (G) Heatmap of tendon-associated genes in FT organoids and 2D TSPCs. (H) Heatmap of chondrocyte-associated genes in FT organoids and 2D TSPCs. (I) Heatmap of chondrocyte adipocyte- associated genes in FT organoids and 2D TSPCs.


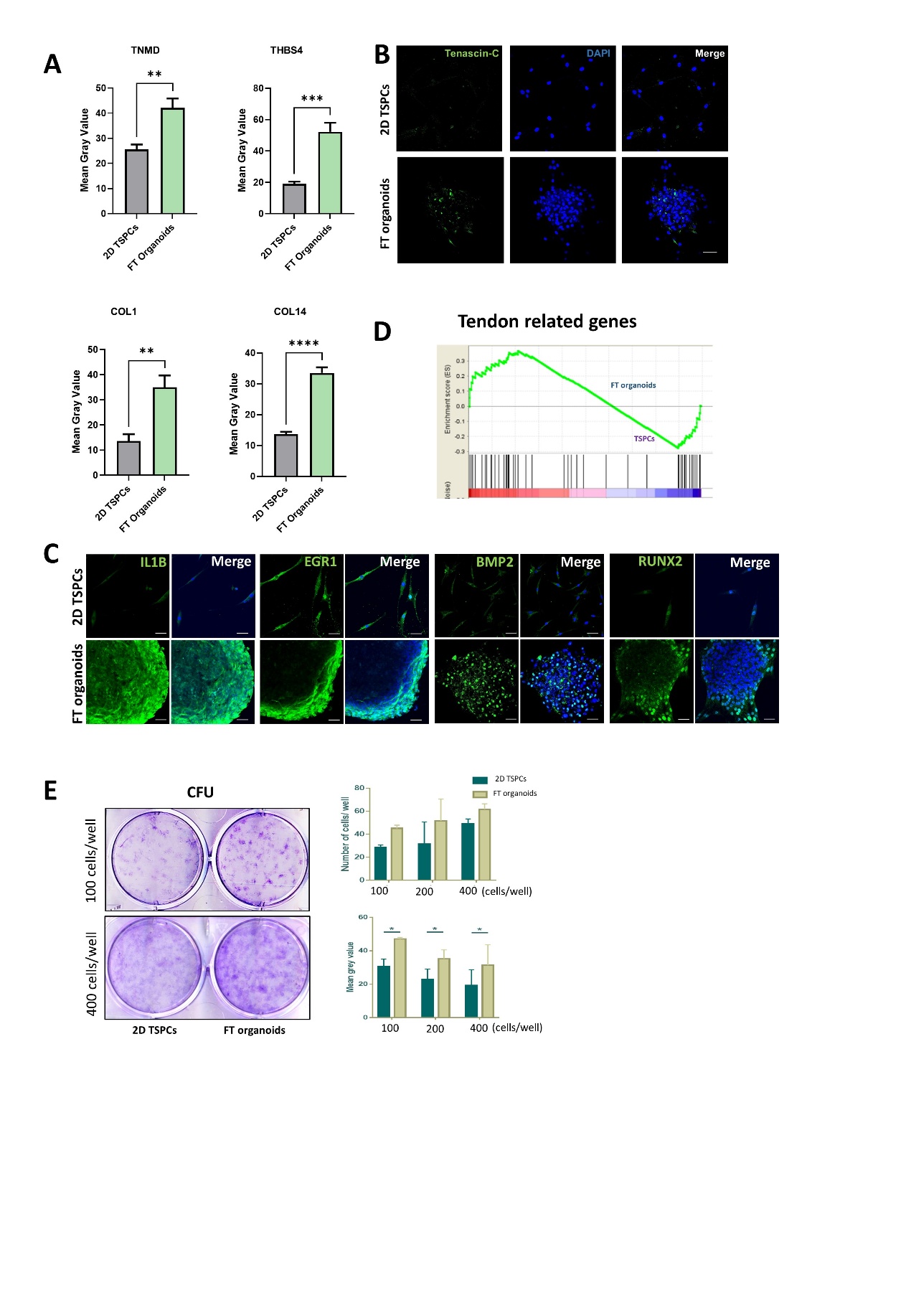


**Fig. S3. Multilineage Differentiation of FT organoids.**

(A) Quantification of mean fluorescence intensity from Fig. 1A. (B) Immunofluorescence staining of Tenascin-C, IL1B, EGR1, BMP2, and RUNX2 (C) in 2D TSPCs and FT Organoids. GSEA analysis of tendon related gene. (D) GSEA analysis of tendon related genes in 2D TSPCs and FT Organoids. (E) CFU assay showing the self-renewal of hTSPCs. Clones were visualized by methyl violet staining. The number of initial cells per well was 100 and 400, from top to bottom (left). The number of clones per well was counted (right, above), and the mean gray value was measured (right, below).


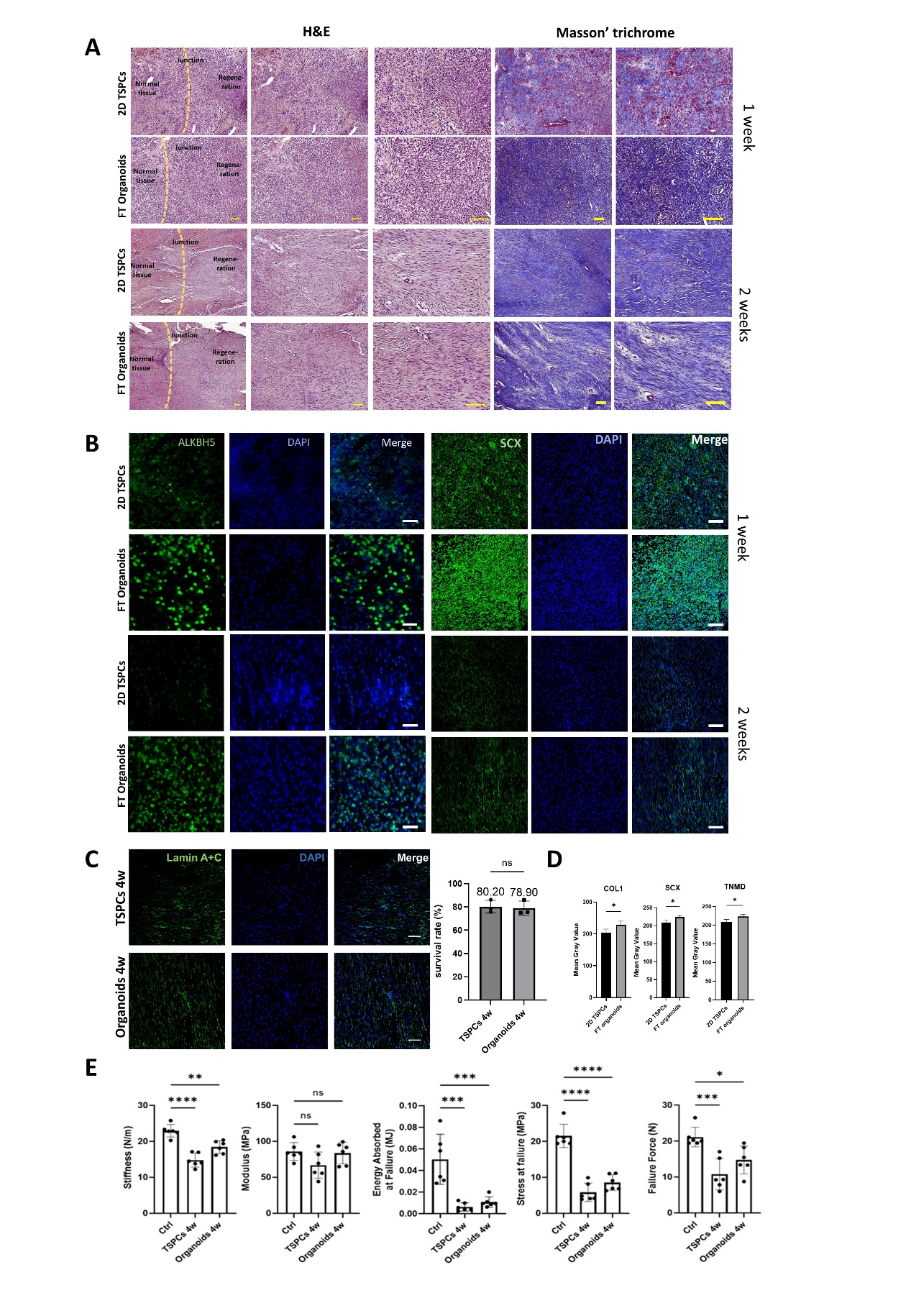


**Fig. S4.** The in vivo reparative potential of FT organoid transplantation for tendon injuries of rats at 1 and 2 weeks post-surgery. (A) Morphology of the repaired tissue site along the axis of the patellar tendon in the FT organoids and 2D groups. H&E staining showing the collagen fibrils at 1 and 2 weeks after implantation. Masson’s trichrome staining showing the deposited collagen at the repaired tissue site and quantitative analyses of the collagen content. (B) Immunofluorescence of tendon-associated genes expressions in FT organoids and 2D TSPCs. (C) Immunofluorescence staining for Lamin A+C and quantification of positive cells in the transplantation area. (Student’s t test)). (D) Mean gray value of COL1, SCX, and TNMD intensity in FT organoids and 2D TSPCs of Figure 4C. (E) The biomechanical properties of the repaired tendon (stiffness, modulus, energy absorbed at failure, stress at failure, and failure force) in the FT organoids and 2D TSPCs groups at 4 weeks after implantation compared with tendon normal control. All data are presented as the mean ± SD. *, p<0.05; **, p<0.01; ***, p<0.001; ****, p<0.0001 (One-way ANOVA). Scale bars 100 μm in (A), 200 nm in (B). Abbreviation: H&E, hematoxylin and eosin.


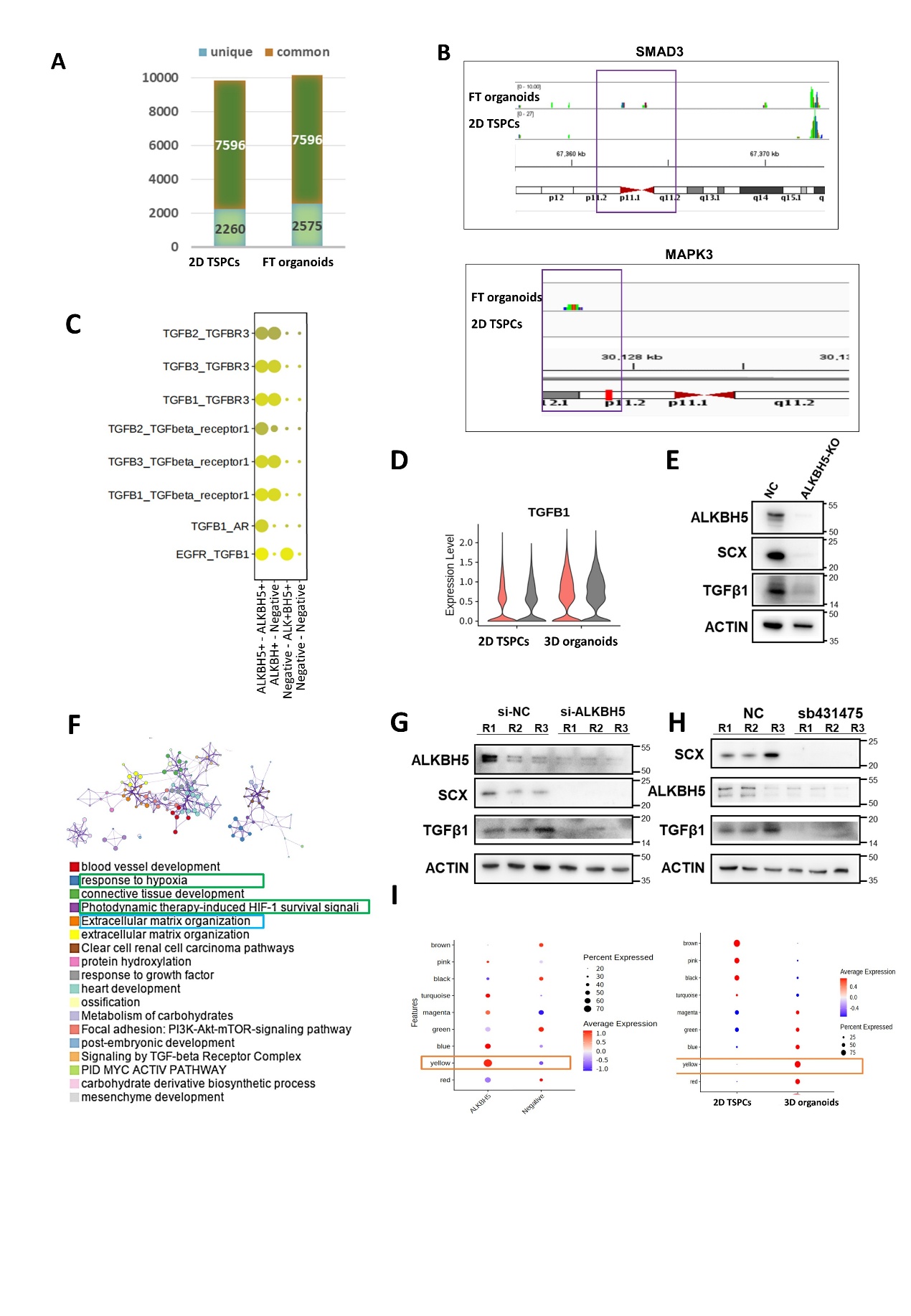


**Fig. S5. Transcriptome-wide identification and analysis of m6A-binding sites in FT organoids and 2D TSPCs.**  (A) Distribution of m6A peaks across the length of mRNA. (B) IGV showed the RNA methylations of genes in TGFβ signaling pathway in FT organoids and 2D TSPCs. (C) Bubble diagram of Receptor-ligand genes in TGF-β signaling pathway between ALKBH5+ and ALKBH5- TSPCs. (D) Violin plot of TGFB1 expressed in the ALKBH5+ and ALKBH5- TSPCs in FT organoids and 2D TSPCs through CellphoneDB. (E) Protein expression of SCX, ALKBH5, and TGFβ1 were detected using tendon cells from ALKBH5-KO mice and control group by Western blot. (F) GO analysis of yellow module by hdWGCNA. (G) Protein expression of SCX, ALKBH5, and TGFβ1 were detected using tendon cells from si-ALKBH5 TSPCs and control group by Western blot. (H) Protein expression of SCX, ALKBH5, and TGFβ1 were detected using tendon cells from TSPCs treated by sb431475 and control group by Western blot. (I) Each module in FT organoids and 2D TSPCs, as well as in ALKBH5+ cells and negative cells.
